# Familiarity Breeds Strategy: In Silico Untangling of the Molecular Complexity on Course of Autoimmune Liver Disease-to-Hepatocellular Carcinoma Transition Predicts Novel Transcriptional Signatures

**DOI:** 10.3390/cells10081917

**Published:** 2021-07-29

**Authors:** Soumyadeep Mukherjee, Arpita Kar, Najma Khatun, Puja Datta, Avik Biswas, Subhasis Barik

**Affiliations:** 1 Department of In Vitro Carcinogenesis and Cellular Chemotherapy, Chittaranjan National Cancer Institute, Kolkata 700026, India; mukherjees960@gmail.com (S.M.); pujadatta.cell@gmail.com (P.D.); 2 Department of Signal Transduction and Biogenic Amines, Chittaranjan National Cancer Institute, Kolkata 700026, India; arpitakar2@gmail.com (A.K.); najma.zhns119@gmail.com (N.K.)

**Keywords:** autoimmune liver disease, hepatocellular carcinoma, HCC transcriptomics, hepatic fibrosis, liver cirrhosis, gene regulatory network

## Abstract

Autoimmune liver diseases (AILD) often lead to transformation of the liver tissues into hepatocellular carcinoma (HCC). Considering the drawbacks of surgical procedures in such cases, need of successful non-invasive therapeutic strategies and treatment modalities for AILD-associated-HCC still exists. Due to the lack of clear, sufficient knowledge about factors mediating AILD-to-HCC transition, an in silico approach was adopted to delineate the underlying molecular deterministic factors. Parallel enrichment analyses on two different public microarray datasets (GSE159676 and GSE62232) pinpointed the core transcriptional regulators as key players. Correlation between the expression kinetics of these transcriptional modules in AILD and HCC was found to be positive primarily with the advancement of hepatic fibrosis. Most of the regulatory interactions were operative during early (F0–F1) and intermediate fibrotic stages (F2–F3), while the extent of activity in the regulatory network considerably diminished at late stage of fibrosis/cirrhosis (F4). Additionally, most of the transcriptional targets with higher degrees of connectivity in the regulatory network (namely DCAF11, PKM2, DGAT2 and BCAT1) may be considered as potential candidates for biomarkers or clinical targets compared to their low-connectivity counterparts. In summary, this study uncovers new possibilities in the designing of novel prognostic and therapeutic regimen for autoimmunity-associated malignancy of liver in a disease progression-dependent fashion.

## 1. Introduction

Autoimmune liver diseases (AILD), a group of immune reaction-mediated chronic liver diseases with a wide range of clinical manifestations, comprises of three principal categories: primary biliary cholangitis (PBC), primary sclerosing cholangitis (PSC) and autoimmune hepatitis (AIH) [1]. PBC is characterized by the presence of anti-mitochondrial antibody (AMA), and is a chronic disease which results from the slow and progressive destruction of the bile ducts leading to the accumulation of the bile in liver, which causes scarring of the liver tissues [2]. PSC is an autoimmune cholestatic liver disorder resulting from the inflamed intra- and extra-hepatic bile ducts [3]. While PBC and PSC primarily involve biliary inflammation, autoimmune manifestations of AIH involve inflammation of the hepatic epithelium, characterized by hypergammaglobulinemia, circulating autoantibodies such as anti-nuclear antibody (ANA), anti-smooth muscle antibody (SMA) and high IgG titers [4]. The etiology of these AILDs remains unknown, but evidence suggests an amalgamation of genetic susceptibility and environmental risks [5], culminating in a loss of immune tolerance leading to the destruction of hepatocytes, which often end up into malignant conditions such as hepatocellular carcinoma (HCC) [6,7,8,9]. Common treatment modalities against AILDs involve immunosuppressant corticosteroid therapies, either as monotherapy or as combination therapy when co-administered with azathioprine [10].

Hepatocellular carcinoma is the fourth leading cause of cancer mortalities of which more than 80% HCC cases are estimated to prevail in Eastern Asia and sub-Saharan Africa, followed by North America and Western Europe [11]. The basic cellular mechanisms underlying the transformation of healthy hepatocytes towards malignancy primarily involve aberrant DNA damage [12], inactivation of cell cycle progression checkpoints [13] and deregulation of cell–cell and cell–matrix interactions [14] apart from various other cellular dysregulations related to the above three processes. Major risk factors for HCC include chronic infection with hepatitis C virus (HCV), hepatitis B virus (HBV), alcoholic fatty liver disease and hematopoietic malignancies, which pose variable effects in risk levels across individual patients [15]. The immune microenvironment of the liver is critically involved in the appearance of HCC signatures associated with most of these risk factors. However, the exact, quantitative role of each component of the immune system in this process is still debatable [16]. As a whole, chronic liver inflammation promotes the early HCC signatures by extensive hepatocyte damage and activation of a local immune response, whereas the immunosuppressive, tolerogenic mechanisms create an anti-inflammatory environment in the later stages, favoring tumor outgrowth by subjection of the cytotoxic immune cells to anergy and senescence [17].

Although the defined mechanism is still unclear, accumulating evidences suggest the AILDs to be key factors participating in HCC development [6,7,8,9]. Briefly, end-stage AILDs often lead to HCC, most likely due to the prolonged, robust hepato-inflammation. Nevertheless, the use of conventional immunosuppressant therapies in the treatment of such patients does not ensure complete remission [18], probably because immunosuppression, in the long run, promotes HCC due to the impairment of the anti-cancer immune surveillance machinery. The most successful treatment method in these cases has, so far, been liver transplantation [19], which also comes with its own demerits such as scarcity of suitable donors, common risks associated with surgical procedures, economic constraints, requirement of long-term post-transplant immunosuppressive therapy, etc. Therefore, it is important to identify the potential risk factors aggravating the transition from AILD to HCC, and pinpoint novel biomarkers associated with those suspected risk factors.

Several factors have been reported to play crucial roles in determining the progression of a liver from the AILD-stage to the malignant stage. Most of the findings have chronicled positive effects of factors such as liver cirrhosis [7,20] and male sex [7,21] in progression towards HCC from an autoimmune inflamed liver. However, molecular intricacies underlining this transition have not been explored in great detail. Such an opaque picture of the molecular regulatory circuitry underlining the AILD-to-HCC transition process highlights the fact that certain grey areas are still there, and yet unidentified molecular players might cause serious trouble during therapy designing. Against such a backdrop, the aim of this study was to collate the genetic signatures associated with the AILDs as well as HCC using advanced in silico approaches to gain a comparative insight into this transition process. Molecules or cascades exhibiting consistent patterns of expression or activity during AILD and HCC were the desired targets since they would offer maximal chances of treatment success as well as minimal chances of off-target effects on course of any therapeutic intervention. Parallel to this, construction of a mechanistic model network based on these common targets to predict the prognostic biomarkers was required for better therapeutic endeavors.

## 2. Materials and Methods

### 2.1. Data Acquisition

A thorough search for gene expression data related to autoimmune liver pathologies as well as hepatocellular carcinoma was conducted on NCBI GEO (Gene Expression Omnibus) DataSets: a global platform for publicly available transcriptomic data. The GSE159676 data, which contains the gene expression profiles of three major autoimmune pathologies of the liver: primary sclerosing cholangitis (PSC, *N* = 12), primary biliary cholangitis (PBC, *N* = 3) and autoimmune hepatitis (AIH, *N* = 3), along with non alcoholic steatohepatitis (NASH, *N* = 7) and healthy control livers (*N* = 6), was selected to analyze the autoimmune liver disease-associated gene expression levels. All autoimmune liver disease samples (PSC, PBC and AIH) were grouped together in a single “AILD” group whenever necessary. The GSE62232 data was chosen for analysis of transcriptome data associated with hepatocellular carcinoma (HCC). This dataset contains 10 non-tumor liver samples and 81 HCC samples. The HCC samples belong to various groups based on pathology-associated criteria such as etiology, Edmondson score and Metavir score. All relevant information about these datasets is available on the NCBI GEO DataSets repository against respective accession numbers. A cohort summary of all samples from both the datasets are available in Table S1.

### 2.2. Data Processing

Normalized expression data were collected from the series matrix files available on NCBI GEO DataSets. Probes were annotated based on manufacturer-provided probe annotation formats. Samples were renamed with their respective group name followed by their replicate numbers. Processed data were uploaded on the iDEP.91 (integrated Differential Expression and Pathway analysis; available on: http://ge-lab.org/idep/; accessed on: 19 March 2021) portal [22] under “Human” as species and “Normalized expression values (RNA-seq FPKM, microarray, etc.)” as data type criterion. Removal of redundant probes was automatically performed by iDEP.91. Log transformation of the values and missing value imputation with gene median values across samples within respective datasets, whenever needed, were carried out on the “Pre-Process” tab of iDEP.91. Qualities of the final data were checked to ensure uniformity in the distributions of expression values across samples (Figure S1A–D). Correlation matrix of all samples was assessed to check the intra-group and inter-group similarities.

### 2.3. Sample Clustering

On the “Heatmap” tab, hierarchical clustergram of the samples were made based on the top 2000 genes with variable expression across sample groups. Euclidean distance with complete linkage was considered, along with centering of genes and samples for heatmap normalization. Multidimensional scaling (MDS)-based clustering of samples was performed on the “PCA” tab. Partition clustering of the transcription factor modules by k-means algorithm was performed on DATAtab online statistical calculator (available on https://datatab.net/statistics-calculator/cluster; accessed on 12 April 2021) using non-tumor liver to F0–F1, F0–F1 to F2–F3 and F2–F3 to F4 as *x*, *y* and *z* axes, respectively. Elbow method was applied to determine the optimum number of clusters for analysis (Figure S6A).

### 2.4. Differential Gene Expression Analysis

Differentially expressed genes between groups were identified using the limma package on the “DEG1” tab. Genes with minimum 1.5-fold change across groups with an FDR cut-off of 0.1 were considered as differentially expressed.

### 2.5. Enrichment Analysis

Over-representation analysis with the common up- and downregulated genes between different autoimmune liver pathologies were carried out on ConsensusPathDB (available on: http://cpdb.molgen.mpg.de/; accessed on 21 March 2021), a web-portal designed for integrating different types of functional interactions between individual cellular entities as well as pathway-based gene sets [23,24]. Pathway-based sets with minimum 5 overlaps with input list and a *p*-value cut-off of 0.01 from “Reactome”, “Kegg”, “Smpdb”, “Ehmn”, “Wikipathways”, “Signalink” and “Biocarta” databases were selected as enriched pathways, and mapped on an interconnected pathway network. Sizes of individual circles represented gene set sizes, while width of the links represented the number of genes shared between two gene sets. The regulatory as well as protein interaction networks of the DEGs were constructed using the “induced network module analysis” function on ConsensusPathDB. Intermediate nodes were allowed as identifiers from inputs. For protein interaction network, only high-confidence, binary interactions were mapped. Gene ontology-based enrichment analysis was performed with common up- and downregulated genes between different autoimmune liver diseases as well as with common up- and downregulated genes between autoimmune liver diseases and HCC (both compared to respective control samples) on FunRich v3.1.4 [25]. Only gene sets with a −log10 *p*-value > 1 were pictorially represented. Gene set enrichment analysis (GSEA) was carried out on the “Pathway” tab of iDEP.91, using the “GSEA (preranked fgsea)” method against gene sets having 15–2000 genes, with a pathway significance FDR adjusted q-value cut-off of 0.2. Functional pathway and interactome-based enrichment analyses with clusters of key transcription factors were carried out on Enrichr (https://maayanlab.cloud/Enrichr/; accessed on 3 July 2021) against “Reactome” and “CORUM” databases, respectively [26,27,28].

### 2.6. Venn Diagram Construction

Venn diagrams of differentially expressed genes across groups from the same dataset were available on the “DEG1” tab of iDEP.91, whereas Venn diagrams of differentially expressed genes across groups from different datasets as well as Venn diagrams of enriched pathways in GSEA were constructed on InteractiVenn [29].

### 2.7. Gene Regulatory Network Construction

The GRN for autoimmune liver disease-associated hepatocellular carcinoma was constructed using BioTapestry v7.1.2 [30,31]. Briefly, a parent model was built using the transcription factors showing altered activity in both AILD and HCC (compared to their respective healthy control groups), and genes upregulated or downregulated in both AILD and HCC datasets falling under at least 3 of these modules (Figure S7). Transcription factors (source nodes) were linked to the downstream genes (target nodes) based on the presence of a gene within a transcriptional module as per “TF.Target.ENCODE” database embedded in iDEP.91. Submodels of this parent model were built for each stage of fibrosis by importing the entire network from the parent model into the submodels. Only those links which came from a source node showing positive correlation between the normalized enrichment scores in autoimmune liver disease vs. healthy liver and fibrosis advanced stage vs. previous stage comparisons, were marked “active” (colored links). All remaining links were marked “inactive” (grey links). Genes with less than 3 links with the transcription factors were not included in the model to increase statistical confidence.

### 2.8. Kaplan–Meier Survival Analysis

Kaplan–Meier survival analysis with the target node genes from the GRN was performed on KM plotter (available on https://kmplot.com/analysis/; accessed on 19 April 2021), an online tool for assessing the impact of genes on survival of patients suffering from various cancer types [32]. The liver cancer RNA-seq platform [33] was used, where patients were split into groups by median expression values of individual genes. Three different kinds of survival: progression free survival (PFS, *N* = 370), disease specific survival (DSS, *N* = 362) and overall survival (OS, *N* = 364) were tested for each gene in the GRN, with no more selection criteria for subjects.

### 2.9. Statistical Analysis

GraphPad Prism v5.0 was used for construction of 2D scatter plots, bar diagrams and all other types of graphical representations. Briefly, normalized enrichment scores (NES) for each of the common transcriptional modules along two comparisons (AILD vs. healthy liver along x-axis, HCC vs. non-tumor liver or advanced stage HCC vs. previous stage HCC along y-axis) were plotted on a 2D scatter plot to identify the correlation between gene expression signatures associated with AILD and HCC. Spearman rank correlation test was conducted to assess the correlation between HCC and AILD signatures across sample groups. Receiver operating characteristic (ROC) analysis was carried out on ROCplotter (http://www.rocplot.org/; accessed on 2 July 2021) with biomarker candidate genes to assess their specificity and sensitivity towards the pathological condition [34]. The AUC (area under curve) value was considered a representative of biomarker quality (the higher, the better). The non-parametric Mann–Whitney test was carried out to compute the statistical significance of the differences between expression values of the candidate genes in early/no fibrosis and advanced fibrosis/cirrhosis conditions. Spearman correlation analysis was performed between the log_2_-transformed expression values of the candidate genes and the log_2_-transformed expression values of canonical markers of liver fibrosis.

## 3. Results

### 3.1. Different Autoimmune Liver Diseases Have an Overlapping Transcriptomic Signature

**Figure 1:**
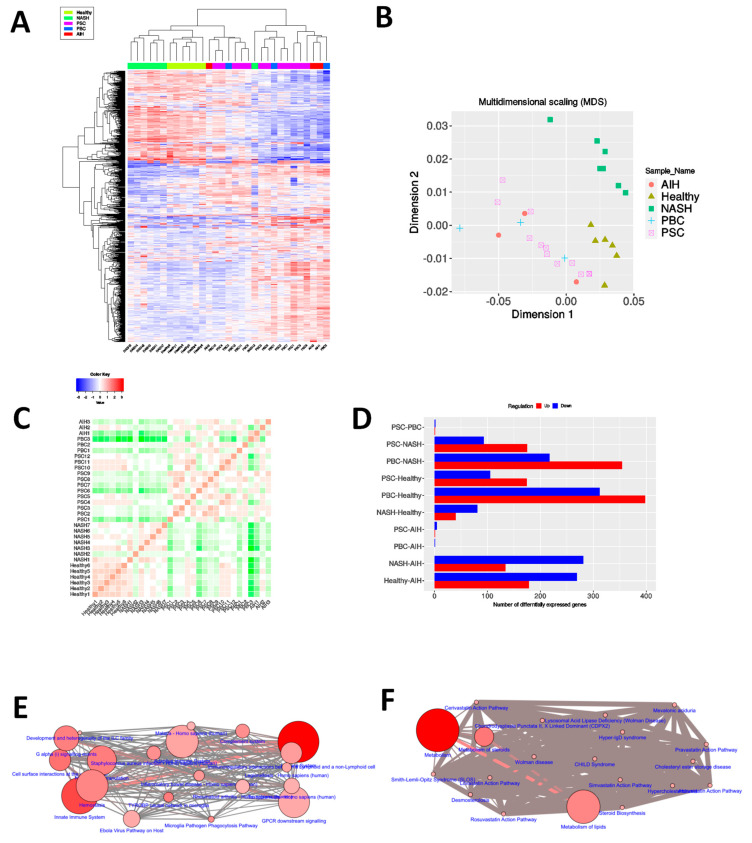
Different autoimmune liver diseases display overlapping transcriptomic signature. (**A**) gene expression heatmap showing hierarchical clustering of inflammatory liver disease samples from GSE159676. (**B**) MDS plot showing unsupervised clustering of inflammatory liver disease samples on a 2D plane. (**C**) correlation heatmap of inflammatory liver disease samples. (**D**) bar diagram showing number of differentially expressed genes (at the top of each bar) between each disease group (**E**,**F**) network of pathways exhibiting maximal enrichment with respect to genes upregulated (**E**) and downregulated (**F**) in all three autoimmune liver diseases, constructed on ConsensusPathDB.

In the modern era of medicine, transcriptome profiling has been one of the most used tools to unfold the molecular complexities of different diseases and disorders [35]. To identify the critical transcriptional changes related to the pathologies of hepatic autoimmune inflammation, the GSE159676 dataset was selected for transcriptome analysis, which contained the gene expression profiles of three major autoimmune pathologies of the liver: PSC, PBC and AIH, along with NASH and healthy control livers. The healthy liver samples showed comparatively tighter clustering on the dendrogram (Figure 1A) as well as the multidimensional scaling (MDS) plot (Figure 1B). Strikingly, the PSC, PBC and AIH samples were found to exist in an overlapping domain within the entire dimensions, both in the dendrogram (Figure 1A) and the MDS plot (Figure 1B). The PSC, PBC and AIH groups exhibited strong correlation among each other in terms of gene expression levels, while showing weaker correlations with healthy controls (Figure 1C). These observations led towards a hypothesis that the three different autoimmune liver diseases share a striking similitude in terms of global transcript levels. Harmonizing with the hypothesis, the number of differentially expressed genes (DEG) among PSC, PBC and AIH was found to be very few, whereas each of the autoimmune disease groups yielded much larger numbers of DEGs when compared to the healthy control groups (Figure 1D, Table S2). Hence, the samples belonging to PSC, PBC and AIH groups were grouped in a single AILD group for further analyses. Common upregulated genes (Table S3) in these three autoimmune liver diseases (Figure S2A) compared to the healthy controls exhibited maximal enrichment in immune cell activation and cell surface–ECM interactions in terms of pathways (Figure 1E and Figure S2B–D, Table S4) as well as regulatory (Figure S2E) and physical interaction (Figure S2F) modules. Common downregulated genes (Table S3, Figure S3A) on the other hand were enriched in metabolism-associated pathways (Figure 1F and Figure S3B–D, Table S4). This metabolically compromised signature of the cells affected with autoimmune liver pathologies were reflected in the gene regulatory (Figure S3E) and physical interaction (Figure S3F) modules.

### 3.2. Progression towards Hepatocellular Carcinoma as Well as Autoimmune Liver Diseases Necessitates Large Scale Cellular Reprogramming

**Figure 2:**
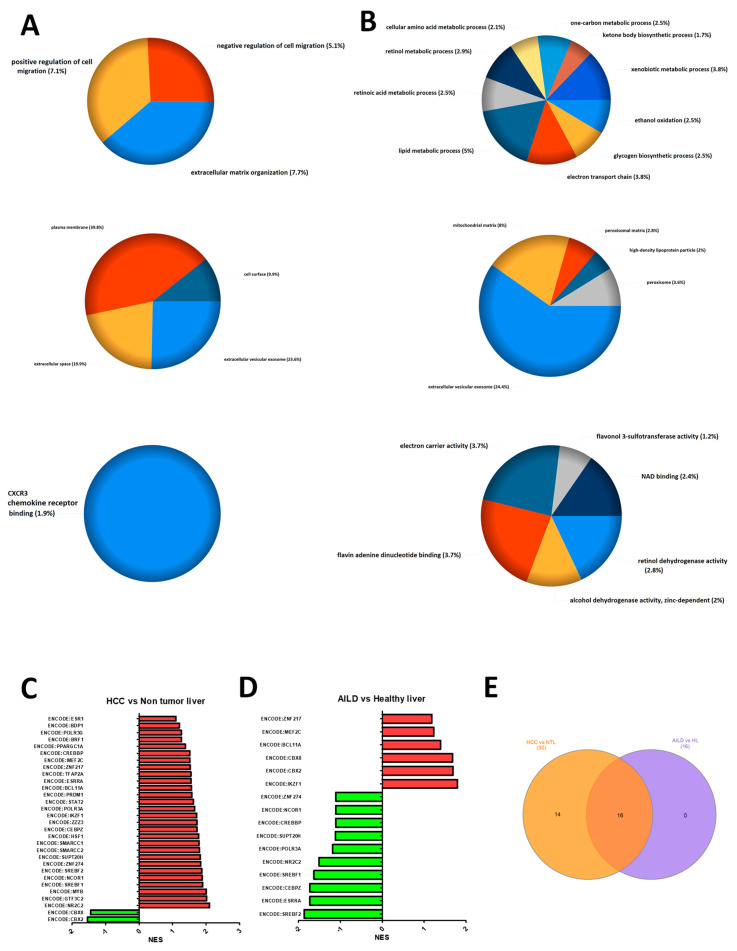
Extensive cellular reprogramming governs progression towards HCC as well as AILDs. (**A**,**B**) gene ontology enrichment analysis with common upregulated (**A**) and common downregulated (**B**) genes between AILDs (GSE159676) and HCC (GSE62232) with respect to healthy/non tumor liver. Panels from top to bottom represent enrichment plots against the GO biological process, GO cellular compartment and GO molecular function, respectively. Analysis and pie chart construction were performed on FunRich v3.1.4. (**C**,**D**) bar plots showing NES of gene set enrichment analysis between AILD vs. healthy liver (**C**) and HCC vs. non tumor liver (**D**) against “TF.Target.ENCODE” database (the green and red colored bars indicate downregulated and upregulated gene sets, respectively). (**E**) Venn diagram presenting the common transcription factor modules (total 16 modules as shown in the overlapping region) enriched in the two comparisons. Orange circle indicates HCC vs. NTL (non tumor liver) and purple circle indicates AILD vs. HL (healthy liver).

Inflamed liver tissues from AILD patients are at a high risk to develop HCC [7]. Pathway enrichment analysis with the DEGs from the GSE62232 dataset showed cell cycle and cytoskeleton reorganization-associated pathways and metabolic pathways, respectively, among the top upregulated and downregulated pathways in HCC (Figure S4, Table S5). To identify cellular events driving a healthy hepatocyte towards HCC through an autoimmune reaction-mediated pathology, differential gene expression analysis was performed in parallel on the two aforementioned gene expression datasets (accession ID: GSE159676, GSE62232), followed by over-representation analysis with the common up and downregulated DEGs (Table S6). The common upregulated genes between HCC and AILD samples compared to healthy livers were maximally enriched in cellular processes related to cell mobility and cell–matrix interactions (Figure 2A), while the common downregulated genes exhibited enhanced enrichment in metabolic cascades, many of which are involved in the redox homeostasis of a cell (Figure 2B). Such resemblance in pathway-level alterations in AILDs as well as HCC led towards the possibility of a metabolically compromised, mobile state of a liver cell on the junction of an AILD-to-HCC transition. Searching for potential regulators of this transition, gene set enrichment analysis with the “TF.Target.ENCODE” database was performed with both the datasets (Figure 2C,D). Expression levels of 16 common transcriptional modules were found to be altered during conversion of a healthy liver towards either of AILD or HCC (Figure 2E). Each module consisted of a master regulator (mentioned in the module name) as well as numerous downstream genes (Tables S7 and S8). Only 25% (4 out of 16) modules showed simultaneous increase or decrease in expression during AILD and HCC, highlighting the fact that AILD does not always lead to HCC [36]. The transcriptomic similarity between NASH and HCC was found to be even lower (4%, 1 out of 25 common modules showing simultaneous increase or decrease during NASH and HCC) when compared to AILD (Figure S5A–B and Figure 2C), indicating the possibility of a greater involvement of these transcriptional modules in HCC development in AILD patients compared to NASH patients. Such a contrast in transcriptomic patterns indicated a clear difference between mechanisms of HCC induction in autoimmune (AILD) and non-autoimmune (NASH) liver inflammatory disorders. However, this calls for further in-depth experimental validation.

### 3.3. AILD-Associated HCC Signatures Develop Independent of Etiology

**Figure 3:**
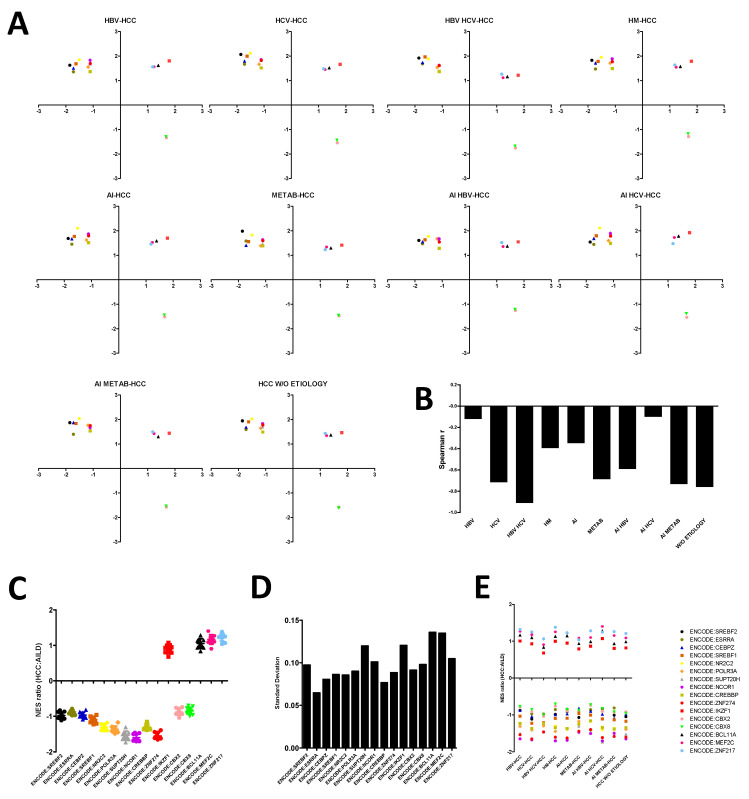
AILD associated HCC signatures across different etiologies. (**A**) 2D scatter plots depicting the enrichment scores of transcription factor modules along AILD vs. healthy liver (*x* axis) and HCC vs. non tumor liver (*y* axis) comparisons for different HCC etiology groups. Enrichment scores were obtained following GSEA against “TF.Target.ENCODE” database on iDEP.91. Moving rightwards along the *x* axis indicates increased expression in AILDs and moving upwards along the *y* axis indicates increased expression in HCC. (**B**) Spearman rank correlation coefficient between AILD and HCC for each etiology group. (**C**) distribution of NES ratio between AILD and HCC for each TF module across all etiology groups. (**D**) standard deviation of NES ratio distributions of TF modules. (**E**) dot plot displaying NES ratio of each TF module for each etiology group. The color and signs of each transcription factor module (for **A**,**C**,**E**) are shown at the right side of (**E**).

Infection of the liver by hepatocarcinogenic pathogens such as hepatitis B or C viruses, intemperate consumption of alcohol, hematological malignancies and hepatic metabolic disorders usually entail the transformation of healthy hepatocytes into HCC [37]. However, there is a dearth of consolidated evidence of any specific etiology of the above being linked to AILD-associated HCC. To address this, HCC samples from the HCC gene expression dataset (accession ID: GSE62232) were grouped separately based on their variable etiologies (HBV: hepatitis B virus, HCV: hepatitis C virus, AI: alcohol induced, HM: hematopoeitic malignancy and METAB: metabolic disorder), followed by GSEA with the “TF.Target.ENCODE” database. Enrichment scores of each of the common transcriptional modules (Figure 2C–E) along two comparisons (AILD vs. healthy liver, HCC vs. non-tumor liver) were plotted on a 2D scatter plot to identify the correlation between gene expression signatures associated with AILD and HCC of a particular etiology. All etiology-based groups of HCC samples showed comparable distributions of the transcriptional modules on the scatter plots with respect to AILD (Figure 3A). Furthermore, HCC samples from no particular etiology-based group showed positive correlation with AILD group based on the expression-behavior of the transcriptional modules (Figure 3B), indicating the lack of coalescence of AILD with any of the reported HCC etiology in terms of developing HCC-associated expression signatures. Relative tendency towards either AILD or HCC (or both) remained largely unchanged for most of the transcriptional modules across samples of different HCC etiologies, apart from a few modules such as ENCODE:BCL11A, ENCODE:MEF2C, ENCODE:IKZF1 and ENCODE:SUPT20H (Figure 3C), which exhibited slightly higher standard deviations than those for other modules across groups (Figure 3D). These modules are most likely to contribute to the majority of the variation in the HCC:AILD transcriptomic correlation across different groups with variable etiologies. In general, groups with different etiologies with stronger negative correlation between AILD and HCC gene expression signatures exhibited less positive HCC:AILD NES ratio for three of these modules, namely ENCODE:BCL11A, ENCODE:MEF2C, ENCODE:IKZF1 (Figure 3E), suggesting a possibly greater importance of these modules in regulating the appearance of HCC signatures in AILD-affected liver tissues.

### 3.4. AILD-Associated HCC Signatures Are Weakly Associated with Histological Signs of HCC

**Figure 4:**
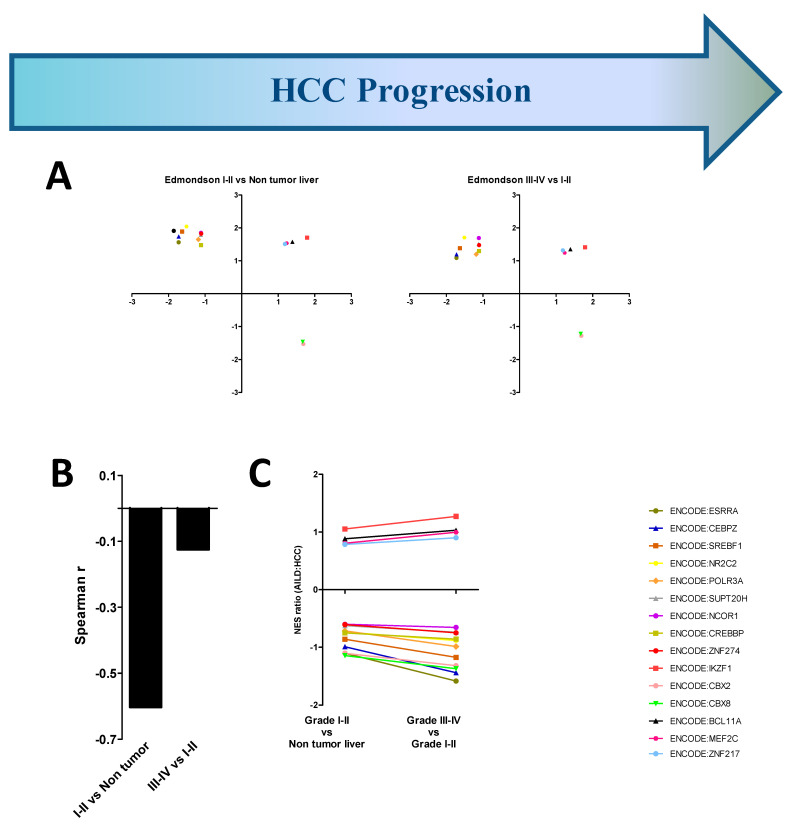
Histological signs of HCC are imperfect correlates of AILD associated HCC signatures. (**A**) 2D scatter plots depicting the enrichment scores of transcription factor modules along AILD vs. healthy liver (*x* axis) and advanced stage vs. previous stage of HCC (*y* axis) comparisons for different HCC etiology groups. Enrichment scores were obtained following GSEA against “TF.Target.ENCODE” database on iDEP.91. Moving rightwards along the *x* axis indicates increased expression in AILDs and moving upwards along the *y* axis indicates increased expression in HCC. (**B**) Spearman rank correlation coefficient between AILD and HCC for each stage of Edmondson–Steiner grading. (**C**) kinetics of shift in NES ratio between AILD and HCC for each transcription factor module across all stages of HCC. The color and signs of each transcription factor module (for **A**,**C**) are shown at the right side of (**C**).

It has been reported that the association between autoimmune liver diseases and HCC become stronger in advanced stages of the disease [7]. Since no particular etiology could be directly linked to the AILD-to-HCC transition, attempts were made to identify how AILD-associated signatures dominate in early-stage and late-stage HCC, which is yet unexplored. The HCC samples were grouped based on their Edmondson–Steiner grade [38], followed by GSEA with the “TF.Target.ENCODE” database and subsequent scatter plot construction. Comparisons between grade I-II samples vs. non-tumor livers as well as grade III-IV samples vs. grade I-II samples showed comparable profiles of the key transcriptional modules (Figure 4A). The magnitude of the negative correlation between AILD and HCC, however, markedly reduced upon progression of HCC towards advanced stages (Figure 4B). Interestingly, no single module showed drastic change in the HCC:AILD NES ration during the transition towards the advanced stage of HCC (Figure 4C), hinting towards the lack of involvement of any particular module alone in establishing AILD-associated-HCC signatures during the advancement of HCC. However, since a net positive correlation encompassing all modules between AILD and HCC was yet elusive, possibilities of larger and more direct contributions from one or more underlying factor(s) accompanying AILD-to-HCC progression could not be overruled. There might as well be a parallel chance of erroneous variability in Edmondson–Steiner grading of the samples, leading to a weakened boundary between grade I-II and III-IV samples, and subsequent infiltration of not-so-advanced stage HCC samples into the advanced stage HCC group; masking the effects of HCC advancement on its transcriptomic correlation with AILDs.

### 3.5. Transcriptomic Signatures of AILD Follow the Fibrotic Patterns on Carcinomatous Livers

**Figure 5:**
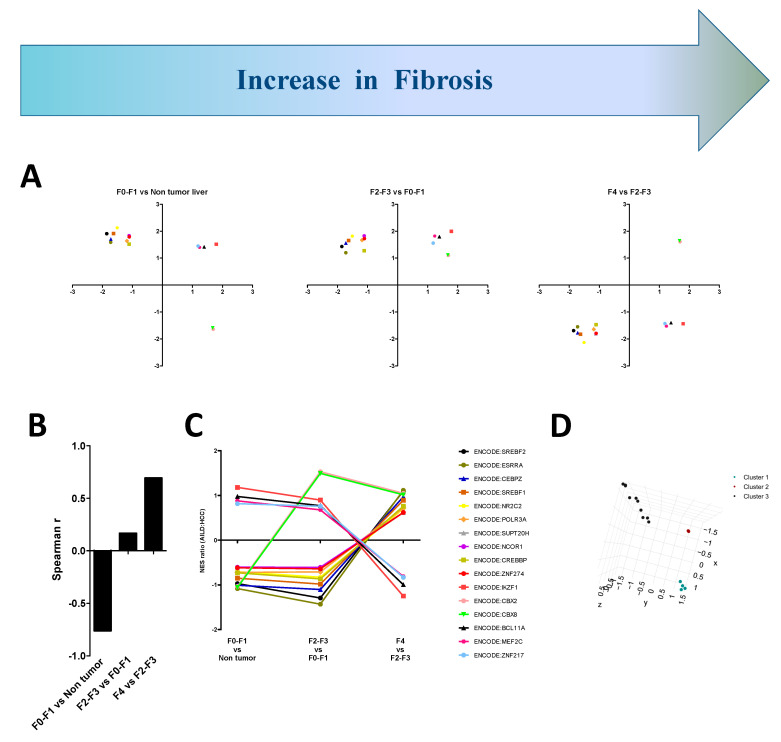
Distinct transcriptomic signatures of AILD are associated with the fibrotic patterns of carcinomatous livers. (**A**) 2D scatter plots depicting the enrichment scores of transcription factor modules along AILD vs. healthy liver (*x* axis) and advanced stage vs. previous stage of fibrosis (*y* axis) comparisons for different grades of fibrosis in HCC. Enrichment scores were obtained following GSEA against “TF.Target.ENCODE” database on iDEP.91. Moving rightwards along the *x* axis indicates increased expression in AILDs and moving upwards along the *y* axis indicates increased expression in HCC. (**B**) bar plot showing Spearman rank correlation coefficient between AILD and HCC for each stage of fibrosis. (**C**) kinetics of shift in NES ratio between AILD and HCC for each transcription factor module across all stages of fibrosis are shown with line diagrams. (**D**) k means cluster plot showing clusters of transcription factor modules in a 3D space based on their NES ratio along progressive stages of fibrosis. *x*, *y* and *z* axes represent F0–F1 vs. non tumor, F2–F3 vs. F0–F1 and F4 vs. F2–F3 transitions, respectively. Distribution of transcriptional modules in each cluster is shown in Figure S6. The color and signs of each transcription factor module (for **A**,**C**) are shown at the right side of (**C**).

Autoimmune conditions of the liver result in a robust immune activation, leading to severe hepatic inflammation and fibrosis [39]. Since fibrosis is an important mediator of HCC progression [40], HCC samples were grouped based on their relative fibrosis levels in terms of the Metavir scoring system [41] before undergoing GSEA with the “TF.Target.ENCODE” database and subsequent scatter plot construction, to delineate the modalities of the regulatory modules in AILD-mediated HCC development through fibrotic progression. Distributions of individual transcriptional modules on different quadrants altered drastically with different stages of fibrosis (Figure 5A). Unlike previous groupings, the ENCODE:CBX2 and ENCODE:CBX8 modules showed drastic upregulation along the HCC axis in the F2–F3 stages in comparison with F0–F1 stages. This upregulation was maintained in the F4 stage as well, whereas the remaining modules, which were upregulated along the HCC axis irrespective of other conditions, showed steep downregulation along the same axis during F4 stage. This trend culminated in a continuous, stout increase in positive correlation between the expression patterns of the modules along the AILD and HCC axes (Figure 5B). Only two modules (ENCODE:CBX2 and ENCODE:CBX8) maintained upregulation along both axes in advanced stages of fibrosis (F2–F3 and F4), while 10 modules (ENCODE:SREBF2, ENCODE:ESRRA, ENCODE:CEBPZ, ENCODE:SREBF1, ENCODE:NR2C2, ENCODE:POLR3A, ENCODE:SUPT20H, ENCODE:NCOR1, ENCODE:CREBBP and ENCODE:ZNF274) underwent strong downregulation in the F4 stage compared to F2–F3, thus imitating their downregulated status associated with AILD (Figure 5C). The remaining 4 modules (ENCODE:IKZF1, ENCODE:BCL11A, ENCODE:MEF2C and ENCODE:ZNF217) exhibited a sharp drop in expression in the F4 stage (Figure 5A). Incidentally, this was the only case where the expression of these modules did not go hand-in-hand between HCC and AILD (Figure 5C). k-means clustering revealed three distinct, well-separated clusters (Figure S6B) of these transcriptional modules (Figure S6C) based on their expression kinetics along the fibrotic progression towards cirrhosis (Figure 5D).

### 3.6. Sequential, Combinatorial Action of Different Clusters of Transcriptional Modules Promotes Fibrosis

**Figure 6:**
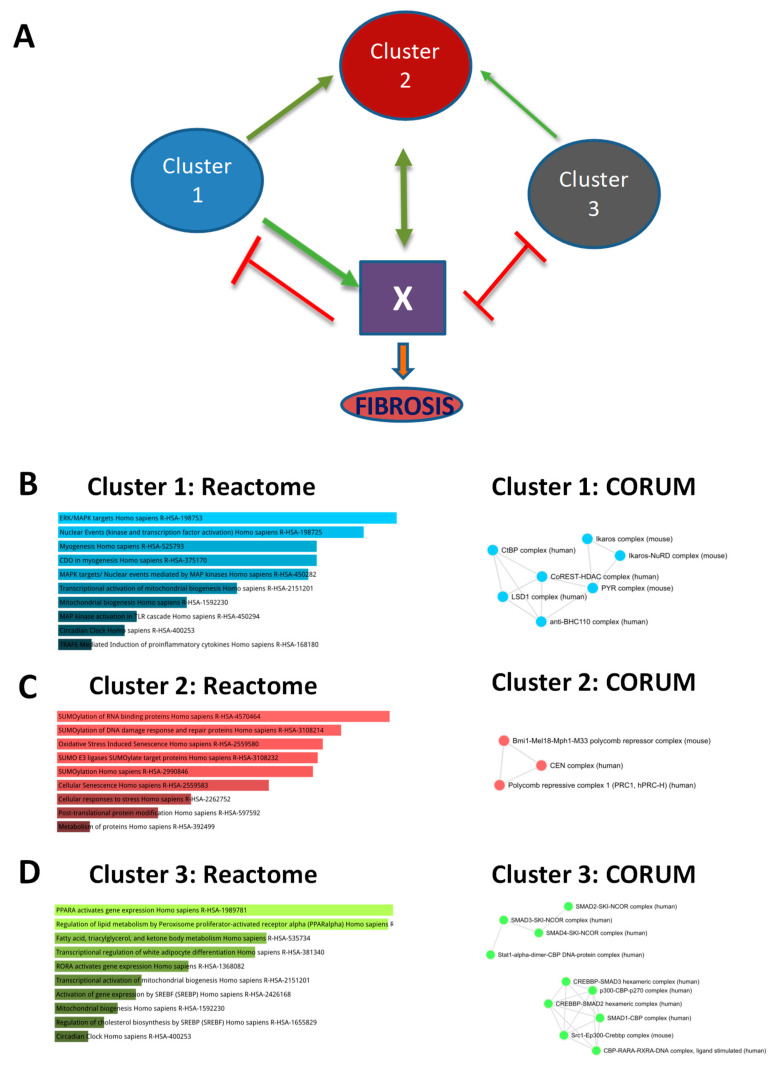
Hypothetical model depicting possible mechanisms of liver fibrosis. (**A**) diagrammatic representation of the proposed dynamic interplay among transcriptional modules during fibrosis. Green and red arrows represent positive and negative regulatory effects, respectively. Clusters 1, 2 and 3 represent groups of transcriptional modules as demonstrated in Figure 5D. (**B**–**D**) enriched pathways from Reactome (**left panels**) and protein–protein interactions from CORUM (**right panels**) for cluster 1 (**B**), cluster 2 (**C**) and cluster 3 (**D**) transcription factors. Enrichment analysis was performed on Enrichr.

To incorporate the interplay of these clusters of transcriptional modules and fibrosis, a possible regulatory model was proposed (Figure 6A). The cluster 1 and cluster 3 transcriptional modules, which were upregulated during the F0–F1 and F2–F3 stages and downregulated in the F4 stage, were hypothesized to act as the “hit-and-run” regulators of the initiation of fibrosis. The cluster 2 modules, which were upregulated only from the F2–F3 stage onwards, were speculated to be required for maintenance of fibrosis, since they maintained positive correlation in terms of expression pattern with AILDs during the stages where HCC-AILD correlation was also positive (F2–F3 and F4). However, since the cluster 3 modules were generally downregulated in AILDs, while the cluster 1 modules showed the reverse behavior, they were expected to play opposite roles in this process. As both these clusters showed a parallel trend of expression, a new factor “X” was brought into the picture to avoid the notion of the clusters repressing or inactivating each other. X, which might be a signature/event/effect or their combination coming from one or multiple sets of genes, was conjectured as a positive regulator of cluster 2 modules and fibrosis, and a negative regulator of both clusters 1 and 3 (Figure 6A). As per the model, the cluster 1 modules would tend to activate X, while the cluster 3 modules would try to suppress X. While this would create a quasi-static equilibrium of X activity, either the cluster 1 or the cluster 3 (or both) modules would collaterally activate the cluster 2 modules, either directly or indirectly. This would push the system towards a stage where the cluster 2 modules would be on and would positively regulate X. As a result, the activity of X would be elevated, leading to a decrease in the activities of cluster 1 and 3. As a loop of positive feedback between cluster 2 and X would keep going on, the system would shift towards cirrhosis, the stage with the highest correlation between AILDs and HCC (Figure 5B). Biological mechanisms underlying the transcriptional activities of the transcription factors from each cluster unraveled the mechanistic basis of the proposed interplay among them (Figure 6B–D). The activities of cluster 1 transcription factors were maximally enriched in pro-inflammatory cascades and myogenic events (Figure 6B left panel, Table S9), which promote the development of an inflammatory signature. Such processes are most likely controlled by these transcription factors as parts of regulatory complexes such as the LSD1 (lysine-specific histone demethylase) complex and Ikaros complex (Figure 6B right panel, Table S9). The cluster 3 transcription factors, on the other hand, showed the best enrichment in events related to lipid metabolism (Figure 6D left panel, Table S9), which plausibly occurs by combinatorial complex formation by these transcription factors with SMADs, p300, STAT1, etc. (Figure 6D right panel, Table S9). Thus, the antagonism between the cluster 1 and 3 modules during the early fibrotic stages are possibly exerted by means of a competition between pro-inflammatory signaling events and cellular lipid homeostasis, where cluster 2 transcription factors skew the balance towards the former by means of extensive posttranslational protein modification-related functions, which they are enriched in (Figure 6C left panel, Table S9). Such processes are possibly exercised by chromatin modulatory complexes such as PRC (polycomb repressive complex) and CEN (centromeric) (Figure 6C right panel, Table S9). Symbiosis among these cellular processes brings forth genetic and epigenetic changes, which culminate in the exertion of X activity and consequent advancement of fibrosis.

### 3.7. The HCC-AILD Common Targetable Gene Regulatory Network Shifts with Progression of Fibrosis

**Figure 7:**
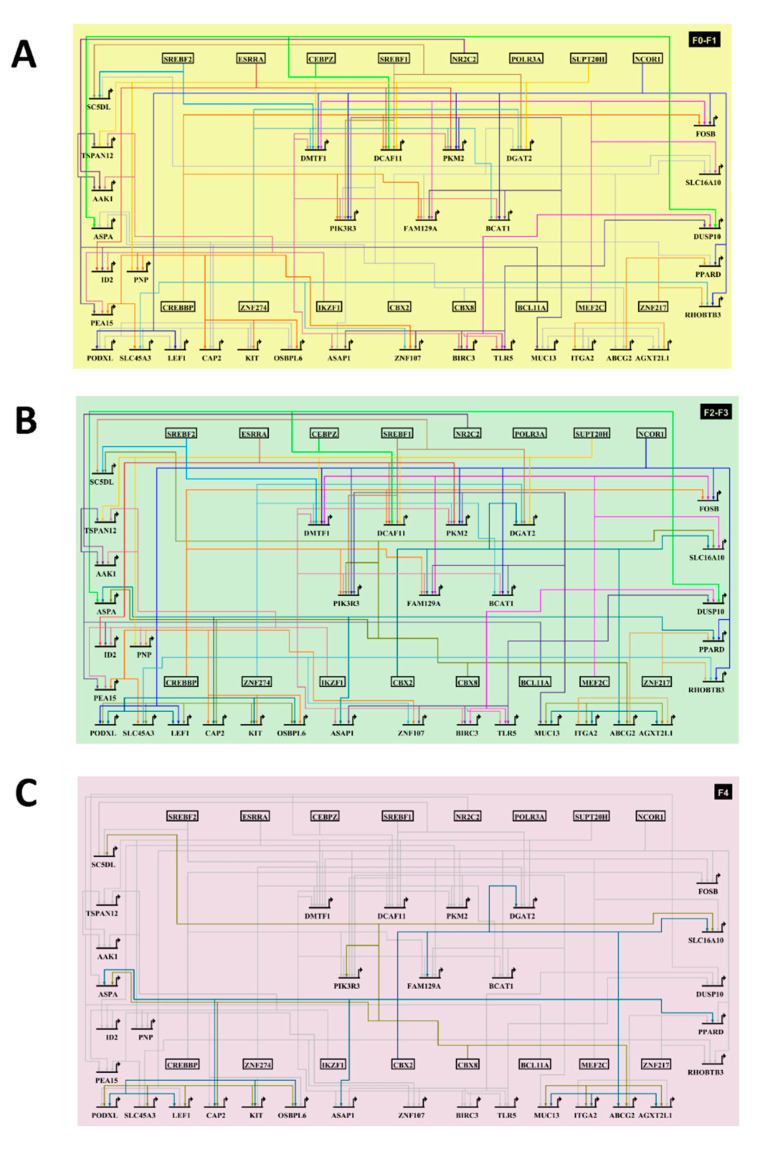
The HCC-AILD associated common gene regulatory network in F0–F1 stage (**A**), F2–F3 stage (**B**) and F4 stage (**C**). Genes in the center are hub genes, having at least four links to the transcription factors (shown in the boxes). All networks are constructed on BioTapestry v7.1.2.

As the predictive model displayed a sequential action of the transcriptional module clusters, it was imperative to understand the molecular circuitry operating downstream of the transcription factors. With this goal, the common DEGs across AILDs and HCC were identified. Next, DEGs belonging to at least one of the transcriptional modules under scrutiny were considered for further analyses. Of all such genes, only 7 genes were part of at least four transcriptional modules (hereafter mentioned as hub genes), while 33 genes (including the 7 genes mentioned before) were part of at least three modules. The gene regulatory network (GRN), made up of these 33 genes and the 16 transcription factors, showed increased transcription factor recruitment on the regulatory loci of the hub genes in the F0–F1 (Figure 7A) and F2–F3 stages (Figure 7B). However, in the F4 stage, most of these regulatory activities were off (Figure 7C), indicating a majority of these transcription factor bindings to be required only for setting up of cirrhotic signatures.

### 3.8. Increased Connectivity in the GRN Manifests into Better Candidacy as Predictive Marker

**Figure 8:**
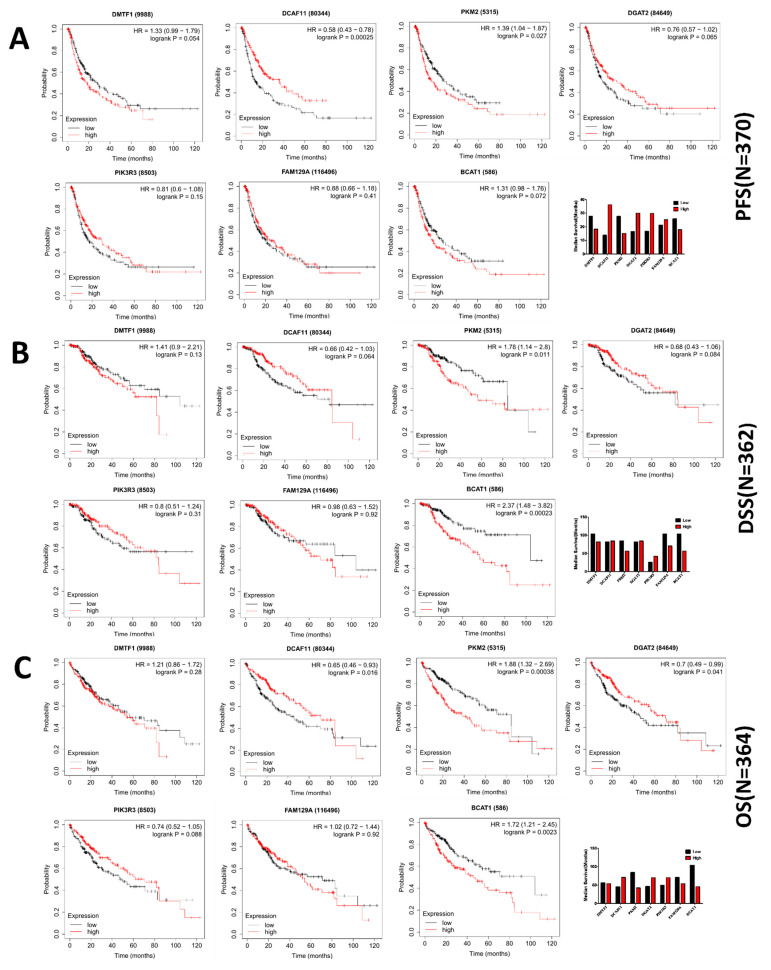
Selected genes in the HCC-AILD GRN show promising prospect as biomarkers as well as therapeutic targets. Kaplan–Meier survival plots of hub genes (genes with ≥4 links) displaying (**A**) progression free survival (PFS), (**B**) disease specific survival (DSS) and (**C**) overall survival (OS). N denotes the number of cases considered for each calculation. Analyzed on KM plotter. Bar diagrams at the bottom right indicate median survival for high and low expression conditions of individual genes.

**Figure 9:**
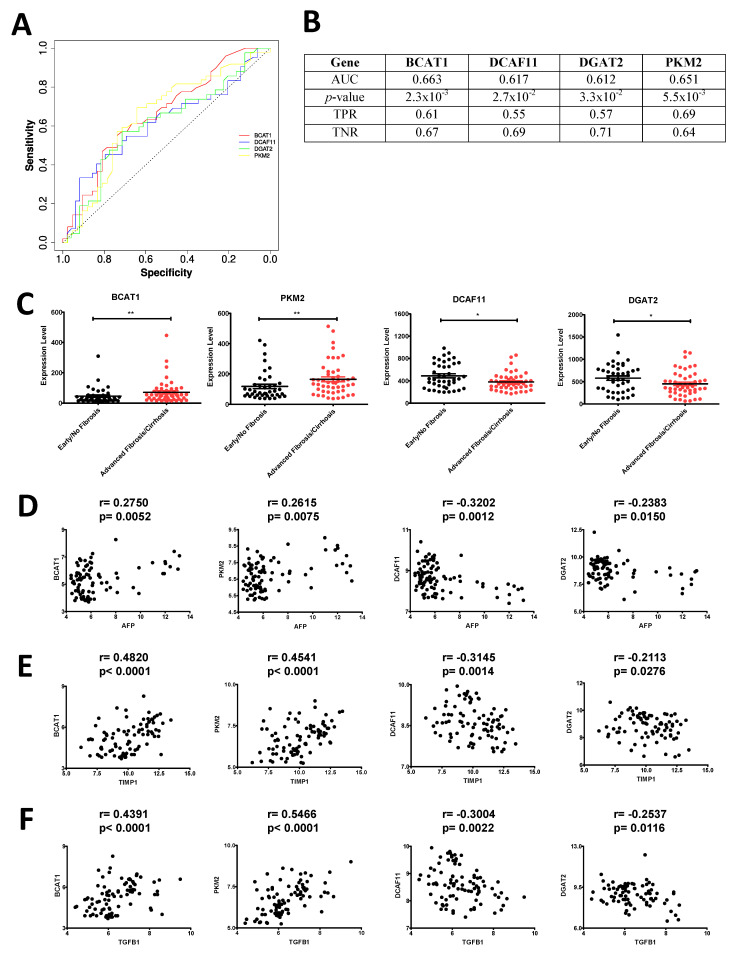
Statistical validation of candidate hub genes as potential biomarkers of fibrosis. (**A**) receiver operating characteristic (ROC) plot of BCAT1, DCAF11, DGAT2 and PKM2 in terms of early/no fibrosis and advanced fibrosis/cirrhosis conditions. The x and y axes represent the specificity and sensitivity of the genes in terms of differences in their expression levels with different degrees of fibrosis. (**B**) attributes of ROC analysis output. AUC; area under curve, TPR; true positive rate and TNR; true negative rate. (**C**) expression levels of BCAT1, PKM2, DCAF11 and DGAT2 in different stages of fibrosis. * and **, respectively, indicate *p* values < 0.05 and <0.01. (**D**–**F**) 2D scatter plots depicting the individual samples with corresponding candidate gene expression level (log_2_ transformed) along *y* axis and AFP (**D**), TIMP1 (**E**) and TGFB1 (**F**) expression level (log_2_ transformed) along *x* axis. Values above each plot denote Spearman correlation coefficient (r) and corresponding *p* value (*p*).

Transcriptional signatures of a disease become clinically relevant only when they qualify as specific, sensitive predictive/prognostic biomarkers. Since the links between the transcription factors and the genes in the proposed GRN (Figure 7A–C) only indicated binding of the transcription factors to the respective regulatory regions without providing any information about the quantitative effects of each transcription factor on the expression of individual genes, it was necessary to assess the relevance of the genes as disease biomarkers. Kaplan–Meier survival analysis was carried out with all the hub and non-hub genes from the network, which would predict the differences in survival of HCC patient cohorts exhibiting respectively high and low expression of each of these genes. The analysis predicted 2 hub genes (DCAF11 and PKM2) in terms of progression free survival (Figure 8A), 2 hub genes (PKM2 and BCAT1) in terms of disease specific survival (Figure 8B) and 4 hub genes (DCAF11, PKM2, DGAT2 and BCAT1) in terms of overall survival Figure 8C) to show significant (*p* < 0.05) differences between the survival kinetics of HCC patients exhibiting low and high expression levels of these genes. PKM2 was the sole candidate exhibiting statistically significant (*p* < 0.05) difference in survival in all three categories, while DCAF11 and BCAT1 were the other candidates which showed statistically significant (*p* < 0.05) differences in survival in at least two categories. The subjects also exhibited strong differences in median survival between high-expressing and low-expressing groups for these genes (Figure 8A–C, bottom right panel). Among the 26 non-hub genes, the number of genes exhibiting significant (*p* < 0.05) differences in terms of progression-free survival, disease specific survival and overall survival were, respectively, 3 (ASPA, ITGA2 and FOSB), 3 (ASPA, ZNF107 and DUSP10) and 5 (ASPA, PEA15, ITGA2, RHOBTB3 and DUSP10). Although these numbers were higher than those from their respective hub-gene counterparts, one must keep in mind the size of their pools (only 7 hub genes compared to 26 non-hub genes), making the fraction of significant non-hub genes minuscule in contrast to that of the significant hub genes (Figures S8–S10). Furthermore, using a slightly relaxed *p*-value cut-off (*p* < 0.1), 4 of the 7 hub-genes (DCAF11, PKM2, DGAT2 and BCAT1) showed provisionally significant differences between the survival kinetics of HCC patients exhibiting low and high expression levels of these genes, in terms of all three genera of survival. Such a *p*-value cut-off is acceptable especially in clinical data-based experiments where cut-throat statistical significance is often compromised due to multiple variables. On the contrary, among the non-hub genes, only 2 out of 26 (ASPA, DUSP10) showed potential as biomarkers based on differential survival of the patients with this relaxed cut-off (*p* < 0.1) exhibiting opposite levels of their expression in all three categories of survival. Such differences in the number of candidates eligible to be used as biomarkers and therapeutic targets between the hub and non-hub groups of genes clearly proved the extent of connectivity in the proposed GRN to act as a robust predictor of biomarker quality. Furthermore, ROC analysis was carried out with non-tumor and F0–F1 samples from the GSE62232 dataset grouped as “early/no fibrosis” group, and the F2–F3 and F4 samples grouped under “advanced fibrosis/cirrhosis” group, to detect the specificity and sensitivity of the candidate hub genes (showing significant difference across groups in all three genera of survival, *p* < 0.1) as disease biomarkers. Results of the analysis showed reasonably good performance by BCAT1 and PKM2 (AUC > 0.65), and satisfactory performance by DCAF11 and DGAT2 (AUC > 0.6) (Figure 9A,B). All four genes exhibited different expression levels between different degrees of fibrosis (Figure S11). BCAT1 and PKM2 were significantly upregulated, while DCAF11 and DGAT2 were significantly downregulated in the later stages of fibrosis (Figure 9C). This pattern was further supported by the statistically significant association between the expression levels of these genes with that of AFP (α-fetoprotein), TIMP1 (tissue inhibitor of metalloproteinase 1) and TGFB1 (transforming growth factor β1), which are known markers of liver fibrosis (Figure 9D–F). This further validated the potential of these candidate genes as fibrosis biomarkers.

## 4. Discussion

Unrestricted aggression of the immune system is a fairly common sequela of all autoimmune diseases, leading to sustained inflammation [42]. The resulting hyper-inflammatory state often favors cancer development [43]. Patients suffering from autoimmune liver pathologies often fall prey to aggressive HCC outcomes [44]. However, because of the immune system acting as a two-edged sword, modes of therapeutic targeting of the AILD or HCC become mutually exclusive, and more often than not mutually antagonistic. Therefore, this study attempted to unfurl the intersecting regulatory checkpoints between the different AILDs and HCC. Among several available -omic approaches, transcriptome analysis was the method of choice given the assumption that significant changes in expression of a set of genes under a certain condition would best represent the functional essentiality of a biological process associated with the genes under consideration in that particular condition, and might aid in identification of novel biomarkers as well as clinical targets.

Immune activation and cell migration-associated processes were upregulated while energy metabolism was downregulated based on the prominent transcriptomic similarities between PSC, PBC and AIH (Figure 1), the three autoimmune mechanism-mediated liver inflammatory diseases. Such patterns are characteristic of an ongoing inflammatory state, with concurrent reshaping of the interplay between the cell and the extracellular matrix as well as the cellular metabolic compartments. The targets of the three diseases being histologically distinct, identification of these common signatures were necessary to exclude any process associated with a specific tissue only and not with the others. Interestingly, analogous signatures were associated with carcinomatous liver tissues as well, indicating these processes to be involved in the transition from AILD to HCC. Since this analysis was primarily focused on the maximally altered processes but the extent of their changes on an absolute scale, a gradient of activation or inactivation of these processes across different grades of the aforesaid transition might be plausible.

The purported involvement of cirrhosis as a mediator of HCC development in AILD patients was mirrored in the positive transcriptomic correlation between AILD and HCC only in case of advanced fibrosis and cirrhosis [7,45]. Additionally, the study annexed an important piece to the puzzle by excavating the kinetics of these transcription factor activities in the pre-cirrhotic and cirrhotic stages. Several biological pathways acting downstream of these transcription factors (Figure 6B–D) have been reported to have decisive roles in the process of hepatic fibrosis. For example, the pro-inflammatory signaling events such as ERK/MAPK pathway, TLR cascades operating downstream of cluster 1 transcriptional modules have been shown to be active during the early fibrotic stages. *Erk2*^-/-^ mice displayed reduced level of liver fibrosis, decreased cell proliferation rate and aggravated immunosuppression in response to fibrosis-inducing diet [46], whereas liver-specific TRAF6 overexpression restored fibrosis in *Traf6*^+/−^ mice, which normally showed compromised fibrosis [47]. On the other hand, lipid metabolic processes regulated by cluster 3 transcriptional modules have been proven to go downhill on course of liver fibrosis. PPARα, the key regulator of hepatic lipid metabolism, was found to exhibit negative correlation with the severity of fibrosis [48]. Additionally, this study itself showed a decline in lipid metabolic activities in AILD as well as HCC-affected livers compared to healthy subjects (Figure 2B). Given the pro-fibrosis role of the SMADs [49], the plausible physical involvement of the cluster 3 transcription factors in SMAD-NCOR complexes, where SMAD activities are repressed [50], suggests the idea of these transcription factors exerting their anti-fibrosis action by suppression of SMAD signaling. The cluster 2 transcription factors operating in the later stages are enriched in physical interactions with chromatin remodelers such as polycomb repressive complex 1 (PRC1), which have been shown to modulate global gene expression levels via histone modification-mediated chromatin remodeling [51]. Interestingly, there is a dearth of existing literature in the involvement of these complexes in the pathology of liver fibrosis. This opens novel opportunities to uncover the mechanistic roles played by the cluster 2 module targets using in-depth epigenetic analyses combined with deep learning or machine learning algorithms.

The intuitive model agglomerated all these information in a predictive network to understand the molecular landscape of autoimmune pathology-mediated HCC development in a single, sequential frame. On an average, genes having more connective links to their upstream transcription factors in the network showed more promise as disease biomarkers as well as therapeutic targets, in terms of their role in disease prognosis as well as their robust correlation trends with existing fibrosis biomarkers. Such better performance by the hub genes as biomarkers compared to their non-hub counterparts strengthened the reasonability and cogency of this study, enabling it to be used successfully in future to understand the course of the disease progression as well as for therapy designing. Furthermore, the genes (especially among the hub genes) showing consistent performance as biomarkers might be empaneled into gene-chips to be used as diagnostic and/or prognostic tools of autoimmune liver inflammation-triggered-HCC on a personalized level.

Extrapolation of the predictive model onto the HCC-AILD correlations based on factors other than fibrosis reveals a deeper insight into the disease mechanism (Figure 3 and Figure 4). The relatively lower magnitude of negative correlation between the enrichment scores based on all transcriptional modules between AILD and HCC in case of Edmondson–Steiner grade III-IV HCC as well as HCC associated with specific etiologies such as HBV and alcohol-plus-HCV might come off as a result of a crucial involvement of fibrosis. However, there must be an additional interplay among certain other factors, which prevents the net correlation score from becoming positive in these cases despite a possibly monumental role played by fibrosis. Identifying these factors and understanding their impact on the dynamics of the transcriptional modules in relation to the course of fibrosis could open new avenues for designing anti-fibrosis therapies, which might be useful in treating all kinds of liver inflammations.

One of the major limitations of this study was the predictive nature of the newly discovered biomarkers. Liver samples of AILD-patients transitioning to HCC are very rare in India, especially Eastern India. Therefore, this study attempted to take on this problem majorly from a bioinformatician’s view, with the use of publicly available resources. Future studies are needed to be carried out on AILD-patient liver samples with or without HCC signatures, and also with different degrees of fibrosis, to validate these in silico findings in an in vitro setting. Another key limitation of this study was the limited sample size of PSC, PBC and AIH groups. Due to the lack of publicly available transcriptomic data corresponding to these diseases with larger sample size, the number of newly identified biomarkers was also limited. However, since most of the AILD-HCC transcriptomic correlations were carried out using all AIH, PBC and PSC samples in a single “AILD” group, the statistical importance of the revelations of this study was not largely affected due to this limited individual sample sizes of AIH, PBC and PSC. Future studies with larger sample sizes might yield even greater number of AILD-HCC common biomarkers, which can then be validated in silico as well as in vitro. Finally, studies should be aimed in future to observe the temporal or stage-based dynamics of the newly identified transcriptional signatures of AILD-HCC in matched patient samples with known status of AILD and HCC, along with their respective fibrosis gradation characteristics and survival timeline, to understand the interplay between the genes and the pathological circumstances in greater detail.

## 5. Conclusions

In conclusion, this study is the first of its kind to identify the global transcriptional regulome underlining the cellular alterations in autoimmune liver pathologies on the way to liver carcinoma. The bioinformatic analyses reveal liver cirrhosis to be a key link between autoimmune liver diseases and hepatocellular carcinoma, explore the dynamicity’s in transcription factor activities along the progressive stages of the diseases and also unravel the predictive common transcriptional signatures associated with these pathologies. Since the findings used for construction of the regulatory network were not biased towards any single autoimmune pathology, the therapeutic applications of the network are expected to be uniformly effective over a broad range of liver and biliary autoimmune diseases.

## Data Availability

All the processed and analyzed data will be made available upon request.

## Supplementary Materials

The following are available online at https://www.mdpi.com/article/10.3390/cells10081917/s1, Figures S1–S11; Tables S1–S9. Briefly, differentially expressed genes were identified from the datasets GSE159676 and GSE62232. Enriched pathways were analyzed based on these DEGs. Pathway-level as well as regulatory networks were constructed before validation of selected DEGs as biomarkers by in silico survival analysis. Figure S1: Quality assurance of processed expression data. Figure S2: Cell–matrix interactions are associated with common upregulated genes in autoimmune liver diseases. Figure S3: Common downregulated genes in autoimmune liver diseases exhibit enrichment in metabolic pathways. Figure S4: Analysis of differentially altered genes/pathways of GSE62232. Figure S5: Overlap in transcriptional signatures between NASH and HCC. Figure S6: k-means clustering of transcription factor modules based on their expression patterns during different stages of fibrosis. Figure S7: Parental gene regulatory network model involving common deregulated transcription factors and target genes in AILD and HCC. Figure S8: Progression free survival (PFS) of HCC patients with respect to variable expression of non-hub genes. Figure S9: Disease specific survival (DSS) of HCC patients with respect to variable expression of non-hub genes. Figure S10: Overall survival (OS) of HCC patients with respect to variable expression of non-hub genes. Figure S11. Expression kinetics of hub genes during (A) AILD and (B) different fibrosis levels of HCC. Table S1: AILD and HCC cohort description. Table S2: DEGS in individual AILDs and NASH. Table S3: Common upregulated and downregulated genes in AILDs. Table S4: Upregulated and downregulated pathways in AILD. Table S5: Upregulated and downregulated pathways in HCC. Table S6: DEGs in AILD group and HCC group. Table S7: Expression profiles of key transcriptional modules in HCC. Table S8: Expression profiles of key transcriptional modules in AILD. Table S9: Pathways regulated by transcription factor clusters.
