# Supplementary material for: Familiarity Breeds Strategy: In Silico Untangling of the Molecular Complexity on Course of Autoimmune Liver Disease-to-Hepatocellular Carcinoma Transition Predicts Novel Transcriptional Signatures
Source: Cells. 2021 Jul 29;10(8):1917. doi: 10.3390/cells10081917 (PMC8394127; doi:10.3390/cells10081917)
Supplement: Supplementary file 1 [file cells-10-01917-s001.zip › Supplementary Files/supplementary figures.pdf]

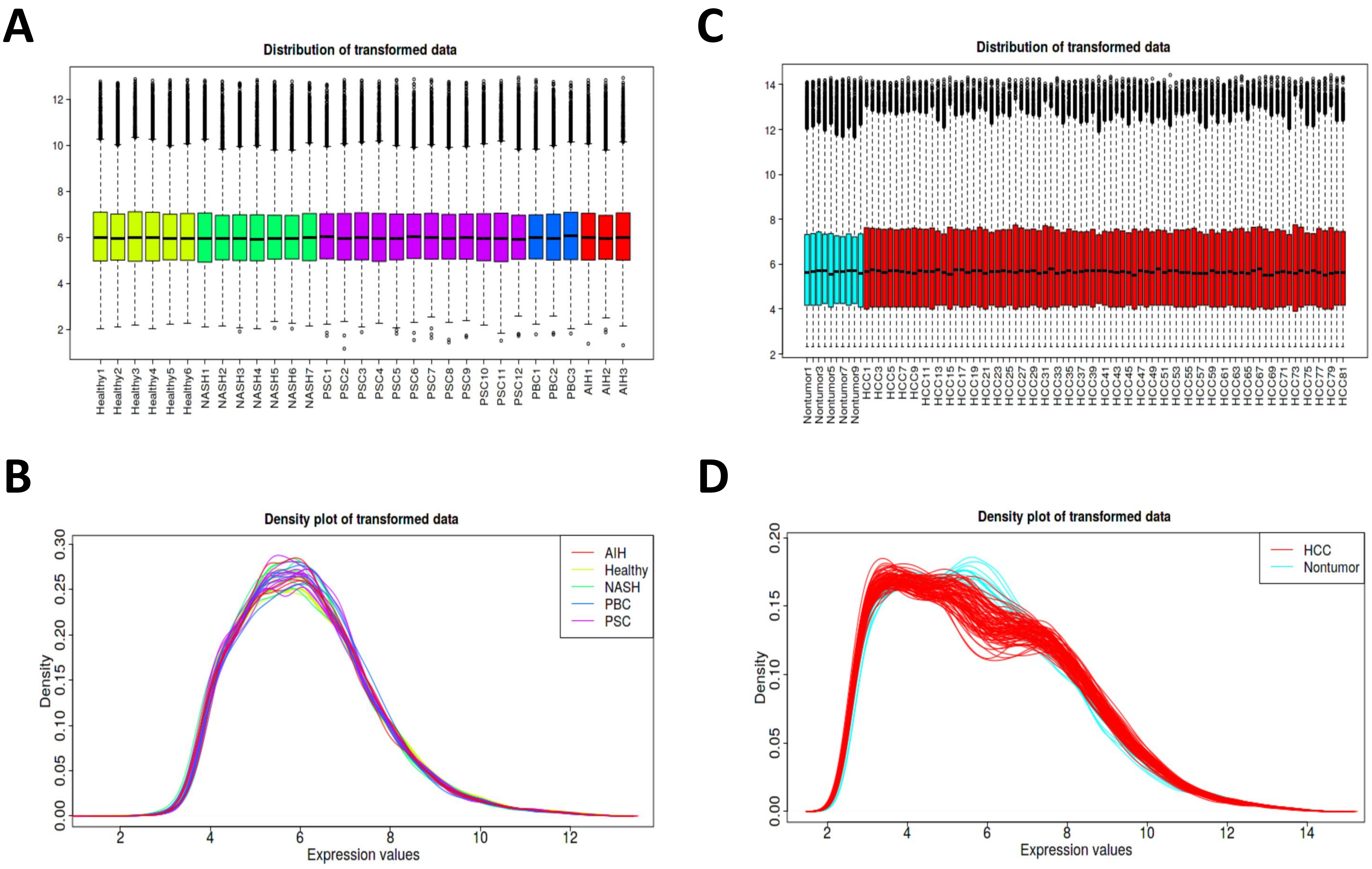

Figure S1. Quality assurance of processed expression data. (**A**) Box plot of expression values from GSE159676. (**B**) Density plot of expression values from GSE159676. (**C**) Box plot of expression values from GSE62232. (**D**) Density plot of expression values from GSE62232.

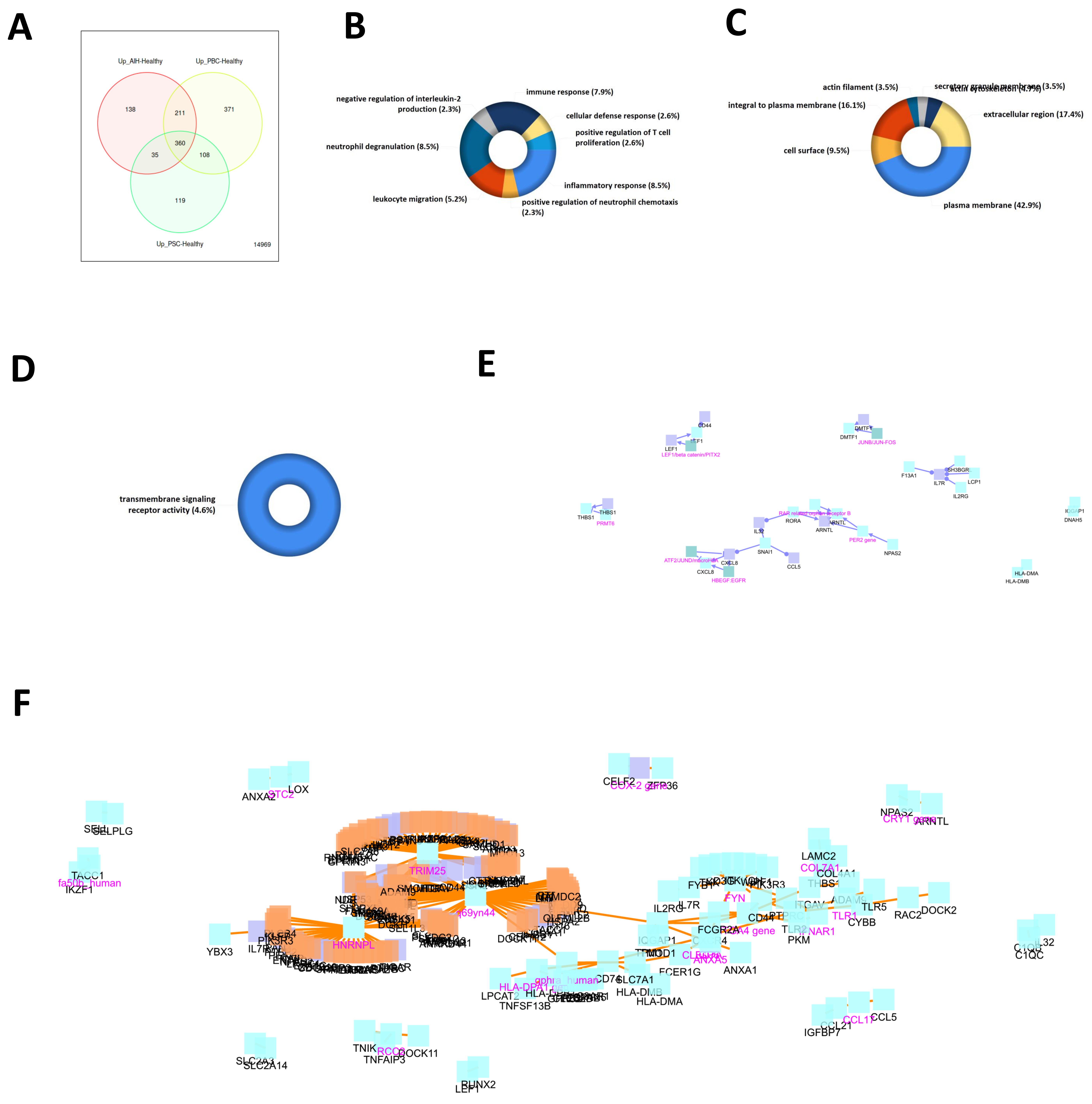

Figure S2. Cell-matrix interactions are associated with common upregulated genes in autoimmune liver diseases. (**A**) Venn diagram of upregulated genes across PSC (green circle), PBC (yellow circle) and AIH (red circle) samples in comparison with healthy liver. (**B-D**) Gene Ontology enrichment results with common upregulated genes, performed in FunRich v3.1.4. Individual doughnut charts represent enrichment results with respect to (**B**) Biological Process (**C**) Cellular Compartment (**D**) Molecular Function. (**E**) Genetic regulatory network of common upregulated genes (constructed using ConsensusPathDB). (**F**) Protein interaction network of common upregulated genes (constructed using ConsensusPathDB).

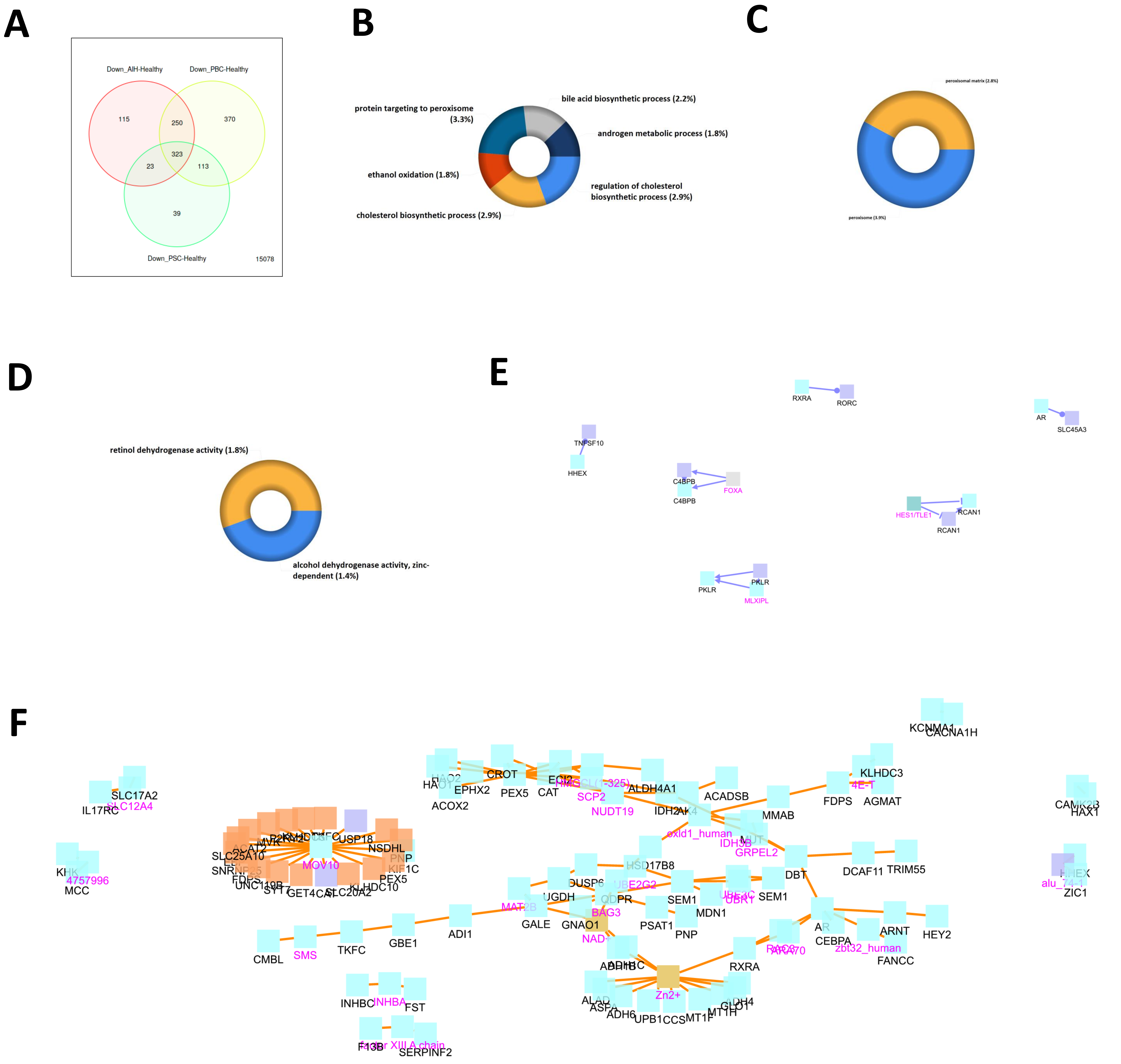

Figure S3. Common downregulated genes in autoimmune liver diseases exhibit enrichment in metabolic pathways. (**A**) Venn diagram of downregulated genes across PSC (green circle), PBC (yellow circle) and AIH (red circle) samples in comparison with healthy liver. (**B-D**) Gene Ontology enrichment results with common downregulated genes, performed in FunRich v3.1.4. Individual doughnut charts represent enrichment results with respect to (**B**) Biological Process (**C**) Cellular Compartment (**D**) Molecular Function. (**E**) Genetic regulatory network of common downregulated genes (constructed using ConsensusPathDB). (**F**) Protein interaction network of common downregulated genes (constructed using ConsensusPathDB).

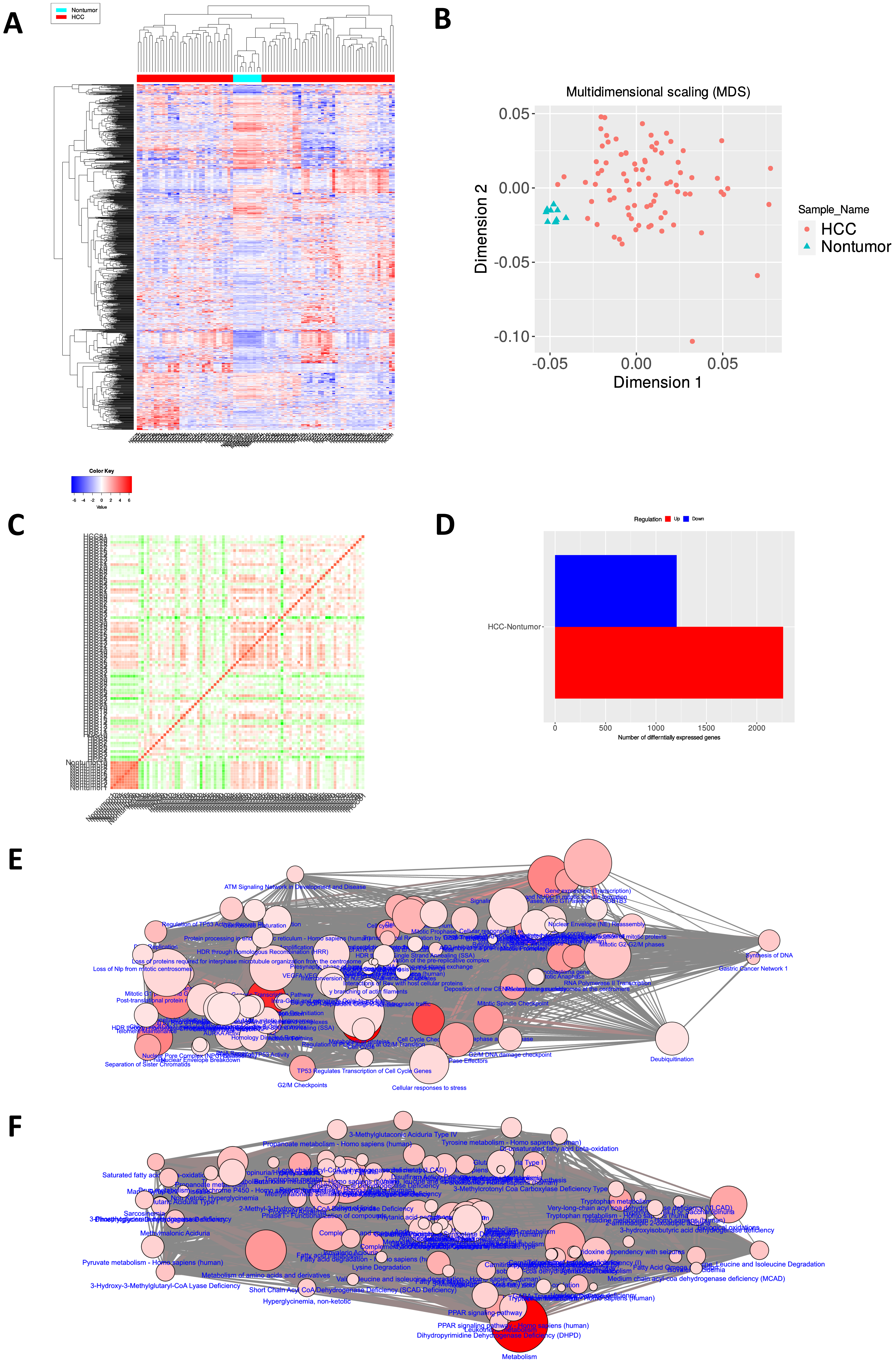

Figure S4. Analysis of differentially altered genes/pathways of GSE62232. (**A**) Gene expression heatmap showing hierarchical clustering of HCC & non tumor liver samples. (**B**) MDS plot showing unsupervised clustering of HCC & non tumor liver samples on a 2D plane. (**C**) Correlation heatmap of HCC & non tumor liver samples. (**D**) Bar diagram showing number of differentially expressed genes between HCC & non tumor groups. (**E-F**) Network of top 100 pathways exhibiting maximal enrichment with respect to genes upregulated (**E**) and downregulated (**F**) in HCC, constructed on ConsensusPathDB.

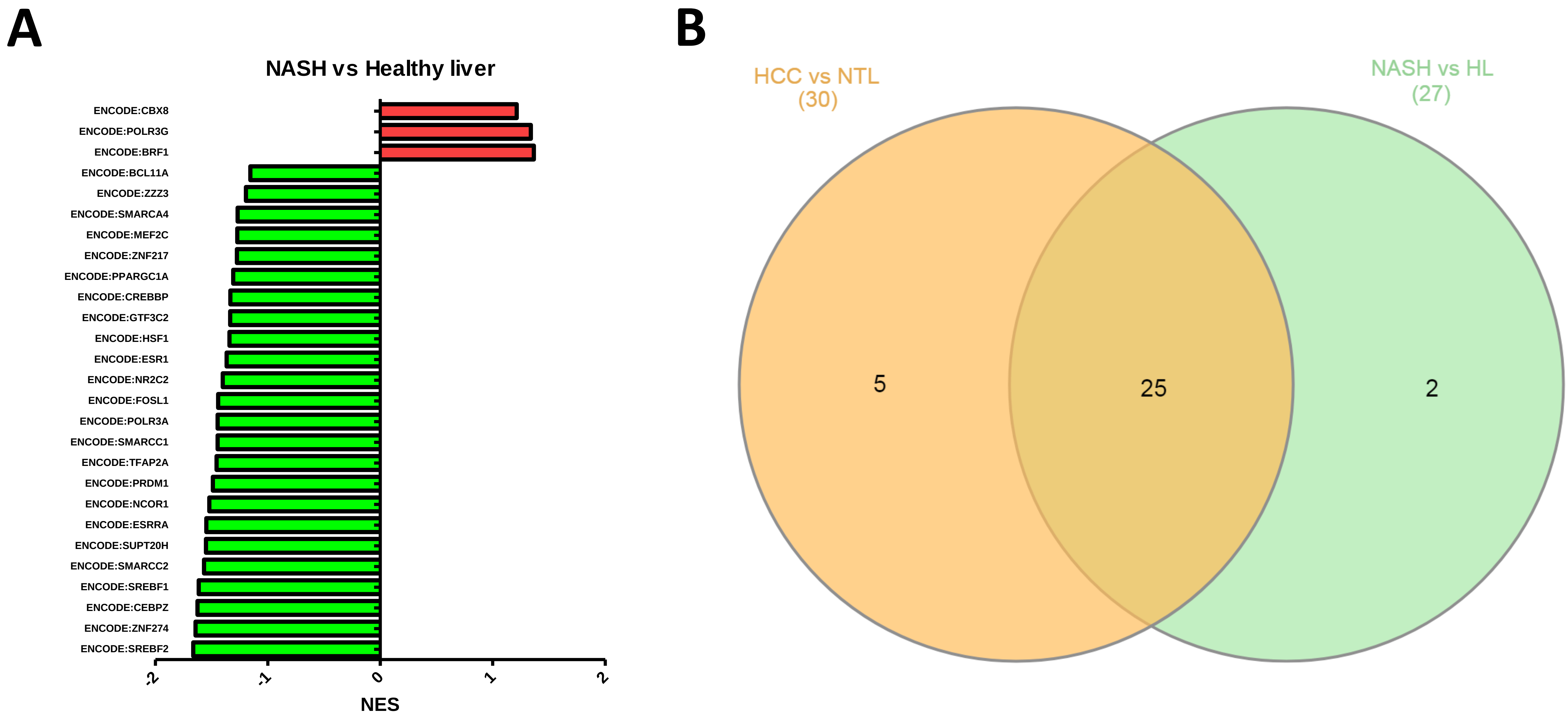

Figure S5. Overlap in transcriptional signatures between NASH and HCC. (**A**) Bar plot showing NES of transcription factor modules from Gene Set Enrichment Analysis between NASH and healthy liver against “TF.Target.ENCODE” database. (**B**) Venn diagram presenting the common transcription factors modules enriched in the two comparisons vs healthy liver**.**

A

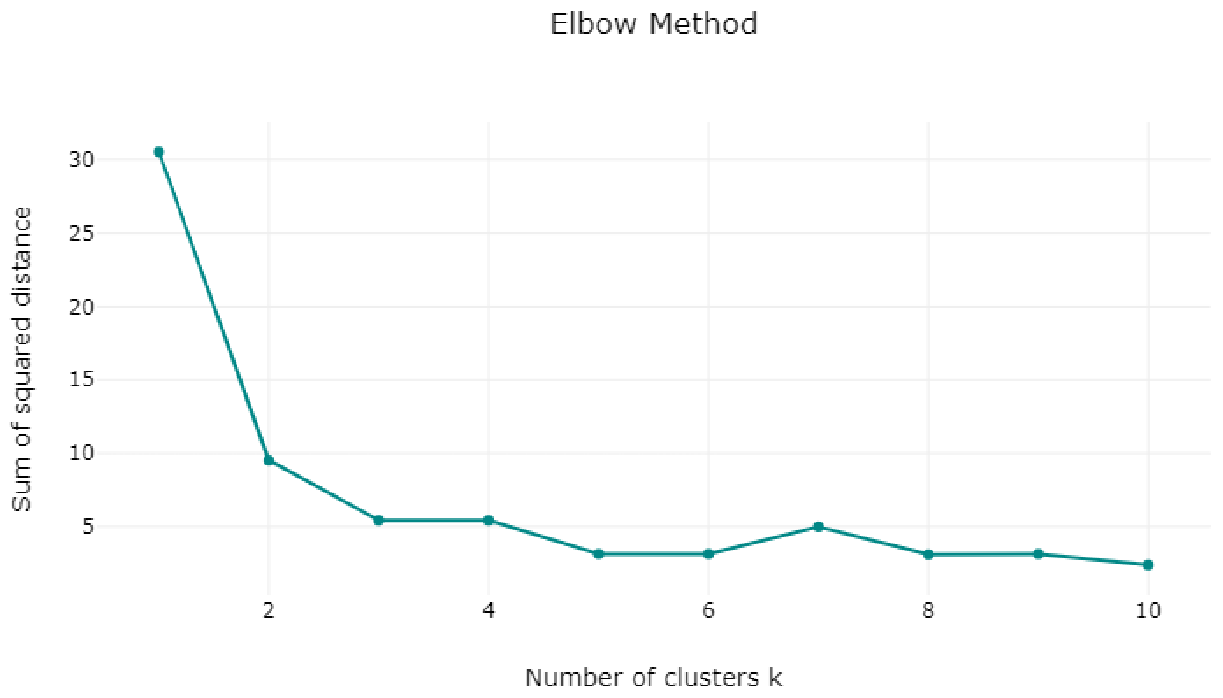

B

Cluster	F0-F1	F2-F3	F4
1	1.06	1.3	-1.06
2	-0.96	0.66	0.97
3	-1.32	-1.19	1.29

C

Transcriptional Module	Cluster	F0-F1	F2-F3	F4
ENCODE: SREBF2	3	-1.03	-0.77	0.91
ENCODE: ESRRA	3	-0.92	-0.7	0.9
ENCODE: CEBPZ	3	-0.99	-0.91	1.03
ENCODE: SREBF1	3	-1.18	-1.02	1.12
ENCODE: NR2C2	3	-1.41	-1.21	1.42
ENCODE: POLR3A	3	-1.39	-1.41	1.38
ENCODE: SUPT20H	3	-1.64	-1.57	1.62
ENCODE: NCOR1	3	-1.65	-1.64	1.61
ENCODE: CREBBP	3	-1.37	-1.15	1.32
ENCODE: ZNF274	3	-1.62	-1.56	1.62
ENCODE: IKZF1	1	0.85	1.11	-0.8
ENCODE: CBX2	2	-0.97	0.65	0.95
ENCODE: CBX8	2	-0.95	0.67	0.98
ENCODE: BCL11A	1	1.02	1.29	-1
ENCODE: MEF2C	1	1.14	1.47	-1.23
ENCODE: ZNF217	1	1.23	1.32	-1.2

Figure S6. k-Means clustering of transcription factor modules based on their expression patterns during different stages of fibrosis. (**A**) Graphical representation of sum of squared distance vs number of clusters. The hinge at number of clusters = 3 denotes this number as the optimal cluster count. (**B**) X-Y-Z coordinates of cluster centres. (**C**) Distribution of transcription factor modules in clusters.

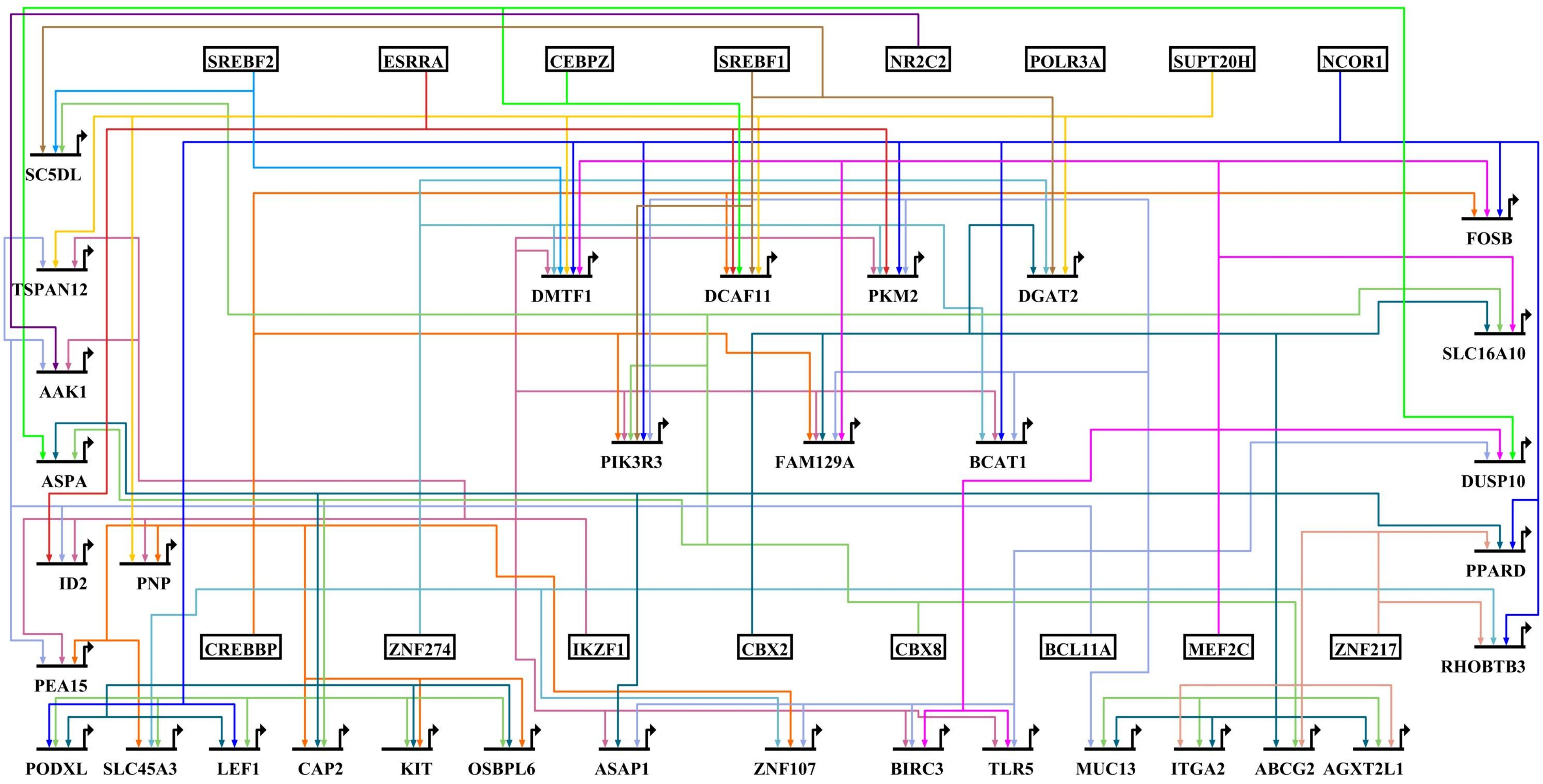

Figure S7. Parental gene regulatory network model involving common deregulated transcription factors and target genes in AILD and HCC. Constructed using BioTapestry v7.1.2.

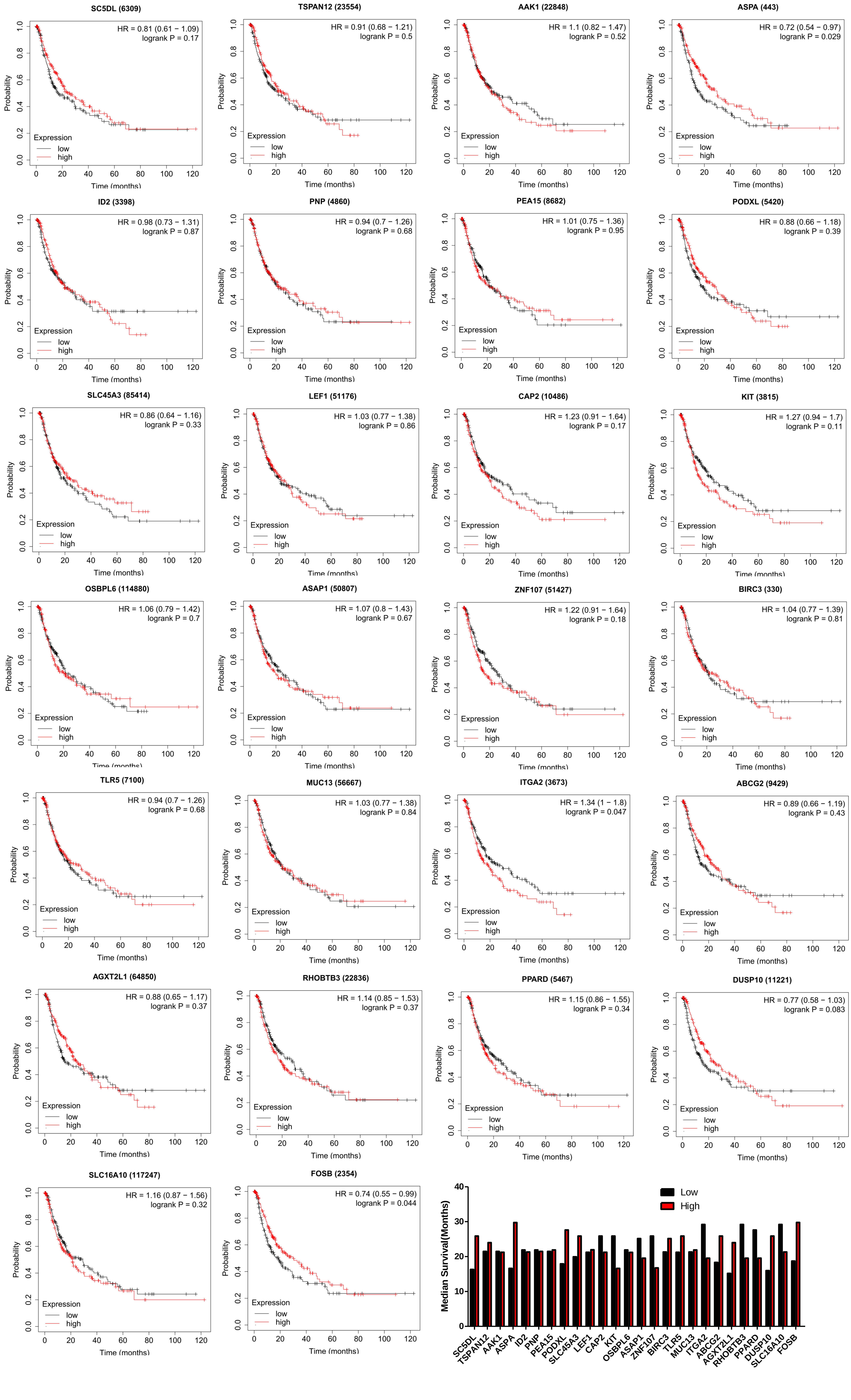

Figure S8. Progression free survival (PFS) of HCC patients with respect to variable expression of non-hub genes. Individual panels show differential survival kinetics. Bottom right panel show median survival for different expression levels of each gene. Analyzed on KM plotter.

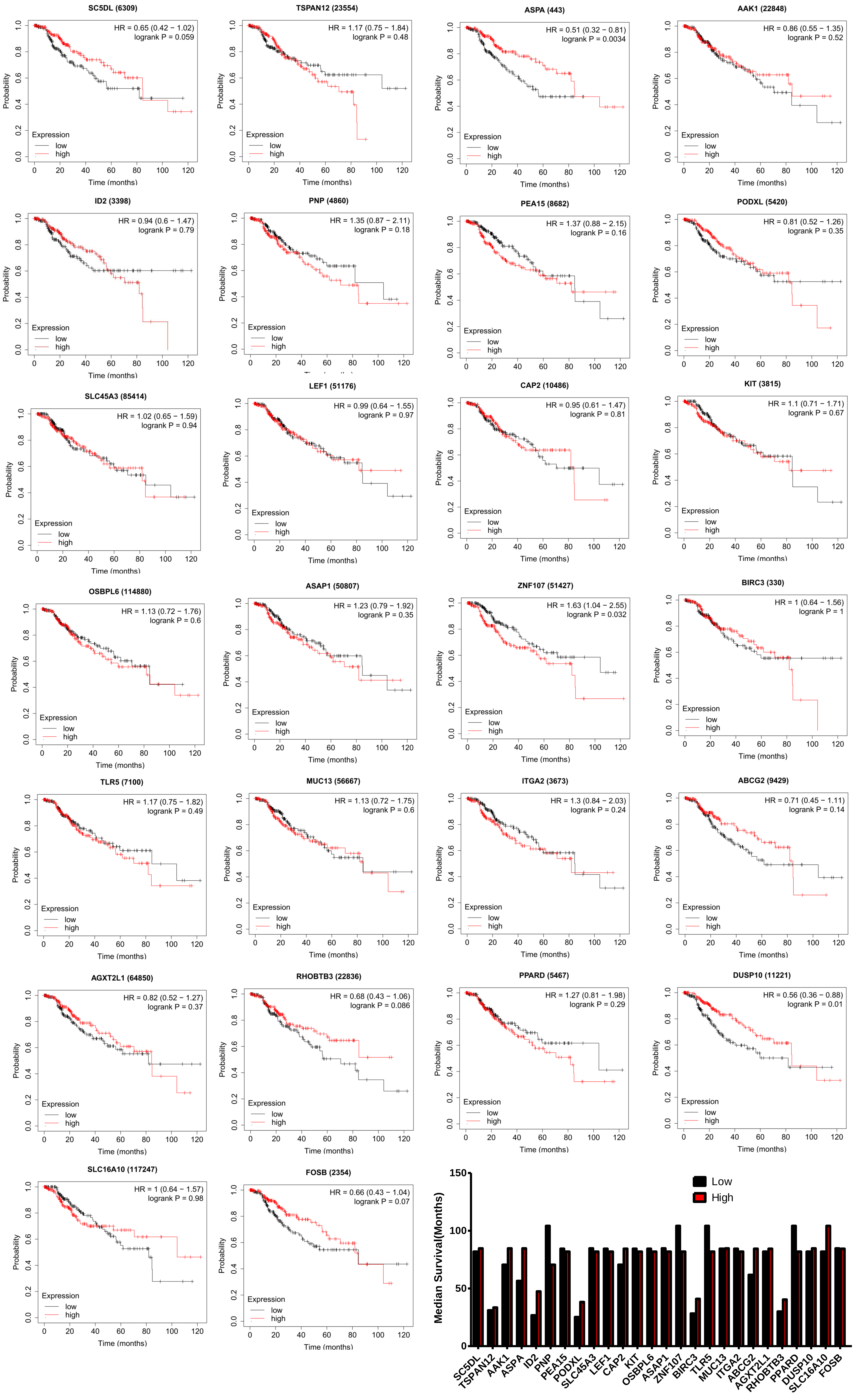

Figure S9. Disease specific survival (DSS) of HCC patients with respect to variable expression of non-hub genes. Individual panels show differential survival kinetics. Bottom right panel show median survival for different expression levels of each gene. Analyzed on KM plotter.

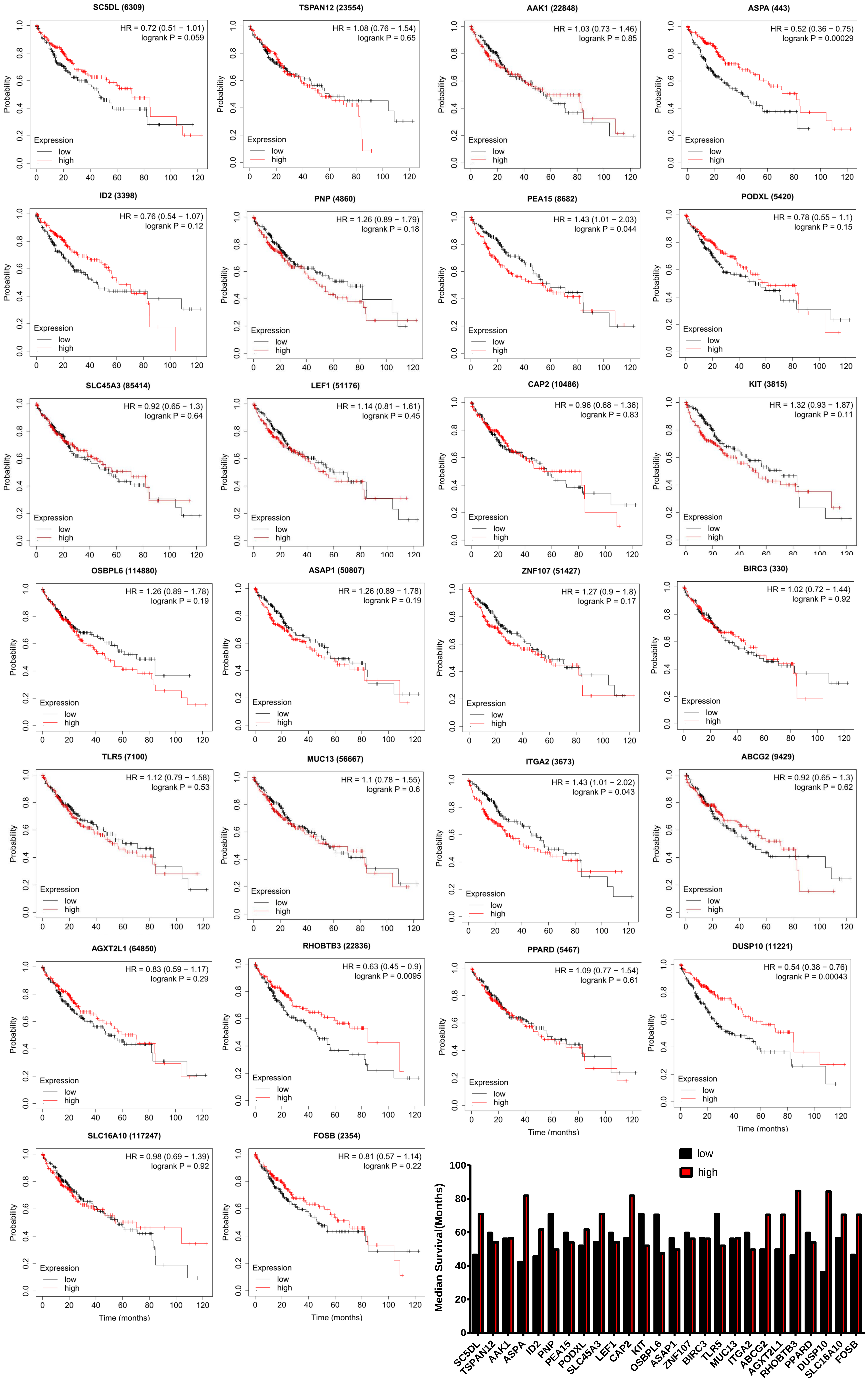

Figure S10. Overall survival (OS) of HCC patients with respect to variable expression of non-hub genes. Individual panels show differential survival kinetics. Bottom right panel show median survival for different expression levels of each gene. Analyzed on KM plotter.

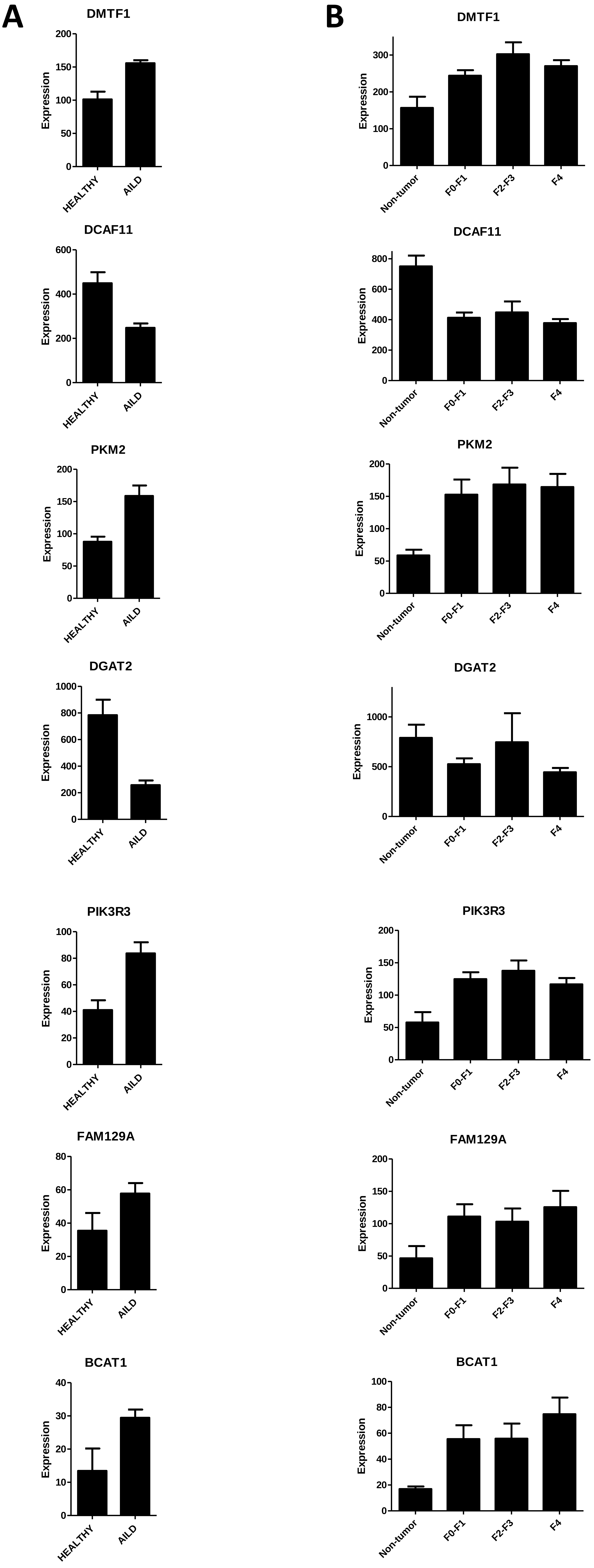

Figure S11. Expression kinetics of hub genes during (**A**) AILD and (**B**) different fibrosis levels of HCC.
